# Pervasive and CpG-dependent promoter-like characteristics of transcribed enhancers

**DOI:** 10.1093/nar/gkaa223

**Published:** 2020-04-27

**Authors:** Robin Steinhaus, Tonatiuh Gonzalez, Dominik Seelow, Peter N Robinson

**Affiliations:** 1 Berlin Institute of Health, Charitéplatz 1, 10117 Berlin, Germany; 2 Institute of Medical Genetics and Human Genetics, Charité – Universitätsmedizin Berlin, Augustenburger Platz 1, 13353 Berlin, Germany; 3 The Jackson Laboratory for Genomic Medicine, 10 Discovery Drive, Farmington, CT 06032, USA; 4 Harvey Mudd College, 301 Platt Boulevard, Claremont, CA 91711, USA; 5 Institute for Systems Genomics, University of Connecticut, 263 Farmington Avenue, Farmington, CT 06030, USA

## Abstract

The temporal and spatial expression of genes is controlled by promoters and enhancers. Findings obtained over the last decade that not only promoters but also enhancers are characterized by bidirectional, divergent transcription have challenged the traditional notion that promoters and enhancers represent distinct classes of regulatory elements. Over half of human promoters are associated with CpG islands (CGIs), relatively CpG-rich stretches of generally several hundred nucleotides that are often associated with housekeeping genes. Only about 6% of transcribed enhancers defined by CAGE-tag analysis are associated with CGIs. Here, we present an analysis of enhancer and promoter characteristics and relate them to the presence or absence of CGIs. We show that transcribed enhancers share a number of CGI-dependent characteristics with promoters, including statistically significant local overrepresentation of core promoter elements. CGI-associated enhancers are longer, display higher directionality of transcription, greater expression, a lesser degree of tissue specificity, and a higher frequency of transcription-factor binding events than non-CGI-associated enhancers. Genes putatively regulated by CGI-associated enhancers are enriched for transcription regulator activity. Our findings show that CGI-associated transcribed enhancers display a series of characteristics related to sequence, expression and function that distinguish them from enhancers not associated with CGIs.

## INTRODUCTION

Promoters and enhancers control the temporal and spatial expression of genes. The core promoter is usually defined as a stretch of 50 base pairs (bp) upstream and 50 bp downstream of the transcription start site (TSS) and serves as a binding site for RNA polymerase II (RNAPII) and its associated general transcription factors (GTFs). Core promoters initiate the transcription of protein-coding and many non-coding genes, but usually have a low basal activity that can be modulated by the proximal promoter and by enhancers (1). Enhancers were classically defined as *cis*-acting DNA sequences that contribute to the spatio-temporal activation of gene expression, function independently of orientation, and are located many kilobases or even megabases distant from their target promoters. Enhancers control gene regulation in a way that is essential for cell- and developmental-specific gene expression (2). Similar to promoters, enhancers contain short DNA motifs that act as transcription-factor binding sites (TFBSs). Binding of transcription factors, modulated by factors such as nucleosome density and post-translational histone modifications, determines the activity of enhancers (3).

Many promoters produce antisense RNAPII divergent transcripts (4,5). Recent findings that not only promoters but also enhancers are characterized by local transcription (6–11) have challenged the notion that promoters and enhancers represent distinct classes of regulatory elements. RNAPII transcribes so-called enhancer-derived RNAs (eRNAs) bidirectionally from enhancer domains enriched in histone H3 monomethylated at lysine 4, i.e. H3K4me1 (12). Additional histone marks characterize enhancer activity, including H3K4me3 and H3K27ac (9,13,14).

eRNAs are typically 0.5–2 kb in length, and their expression levels tend to correlate with the cis-regulatory activity of their template enhancers (15). The functions of eRNAs have not been comprehensively elucidated, but available evidence suggests that eRNAs may function by a variety of molecular mechanisms. For instance, at least some eRNAs may be able to facilitate spatial interactions between enhancers and promoters and thereby enhance transcriptional activation (16). eRNAs can bind to CREB binding protein (CBP) and thereby stimulate its histone acetyltransferase activity; CBP binding is characteristic of enhancers, and eRNA binding can lead to changes in the histone acetylation mediated by CBP (17). eRNAs can interact with the co-activator complex Mediator and thereby affect gene transcriptional activity (18), and can interact with genome architectural proteins such as cohesin (19). However, in one case knockdown of an eRNA had no effect on the transcription of its target gene (20), supporting the idea that in some cases, at least, eRNAs may be a by-product of enhancer-bound RNAPII without independent biological function (15,21).

In this work, we investigate correlations of core promoter elements (CPEs), CGIs, and transcription-factor binding events with functional characteristics of transcribed enhancers. CPEs are binding sites for general transcription factors (also called basal transcription factors), which recruit RNAPII (1,22–24). CPEs display localized overrepresentation in promoters, meaning that CPEs can be represented by position-specific weight matrices that are positionally correlated with the TSS (25). Previous work has confirmed localized overrepresentation of TATA, Inr, DPE and BREu (BRE upstream of TATA). In addition, it has been proposed that specific combinations of CPEs may mediate distinct categories of preinitiation complex-DNA interaction as reflected by statistically significant co-occurrences of individual CPEs (26). For instance, TATA-less genes have a higher than expected proportion of core promoters with strict Inr elements (27) and are also commonly associated with CGIs (28). Individual CPEs have been associated with other genomic characteristics; for instance, genes whose promoters contain TATA boxes often tend to be more tissue specific than those that do not (29). CPEs have yet to be comprehensively investigated in enhancer sequences.

CAGE (Cap Analysis of Gene Expression) sequencing was used by the FANTOM consortium to profile the transcriptomes of a large panel of human tissues and cell types, demonstrating the existence of over 60 000 bidirectionally transcribed enhancers that gave rise to mainly nuclear and non-polyadenylated RNAs (8). Transcription of these enhancers was shown to precede transcription of target promoters in cellular differentiation or activation (30). These results led to the hypothesis that promoters and enhancers can be considered to be a single class of element whose function is dependent on RNAPII-mediated transcription and whose functional output is determined by the surrounding sequences and the genomic context (10,31). Indeed, promoters and enhancers contain partially overlapping sequence motifs that presumably explain at least some of the functional commonalities and differences (32–34).

In this work, we show that CPEs demonstrate statistically significant localized overexpression in transcribed enhancers. Furthermore, we demonstrate that promoters and transcribed enhancers share a number of characteristics whose magnitude in both cases correlates with the presence or absence of a CGI.

## MATERIALS AND METHODS

### Data sources

The work presented in this manuscript is based on promoter definitions taken from the Eukaryotic Promoter Database New (EPDnew) dataset, version 006 (35) (Hs_EPDnew_006_hg38.bed). This dataset represents a compilation of 29 598 promoter sequences for which the TSSs have been determined experimentally.

To investigate transcription of promoters and enhancers, we used CAGE tag data from the FANTOM5 project (36). 1829 CAGE libraries were used, including 188 tissue, 564 primary cell, 271 cell line, 785 time-course and 21 fractionated cell libraries (37). The FANTOM5 consortium leveraged the CAGE data to identify transcribed enhancers based on divergent transcription from the enhancer. About 95% of RNAs originating from enhancers were unspliced and typically shorter than mRNAs. Enhancers showed no evidence of associated downstream RNA processing motifs, and very few enhancer RNAs overlapped exons of known protein-coding genes or lincRNAs (8).

### Identification of CPEs

Position weight matrices (PWMs) were computed for twelve CPEs. A PWM of length ℓ assigns each oligonucleotide of length ℓ a matching score $x=\sum\nolimits _{i=1}^{\ell }w_{bi}$, where $w$_*bi*_ is the weight of base *b* at column *i* of the matrix. The weights $w$_*bi*_ were computed relative to the log-normalized base frequencies per position of experimentally derived binding sites (25) (Supplementary Table S1). We called a CPE to be present at the location of the oligonucleotide if the score exceeded a matrix-specific cutoff value (Supplementary Table S2).

### CGIs

In the human genome, CpG dinucleotides are present at about 20% of the frequency that would be expected based on the overall GC-content. The depletion of CpG dinucleotides in the human and other mammalian genomes is due to the increased mutability of methylcytosine within CpG dinucleotides. Stretches of GC-rich (∼65%) sequence in which the observed frequency of CpG dinucleotides is close to the frequency that would be expected based on the individual frequency of G and C bases are termed CpG islands (CGIs). CGIs are associated with the upstream region of many genes generally covering all or part of the promoter and displaying an average size of ∼1 kb (38,39).

To identify CGIs in this study, a 100-nucleotide window was shifted in 1 bp intervals across the promoter sequences from position [−200, −100) relative to the TSS to (+100, +200]. The percentage GC-content and CpG observed/expected ratio

$$\begin{equation*} \frac{\textrm {Number of CpG}}{\textrm {Number of C}\times \textrm {Number of G}}\times 100 \end{equation*}$$

were calculated per window. A promoter or enhancer was considered to be associated with a CGI if all consecutive windows within a region of at least 200 bp had a GC-content ≥50% and a CpG observed/expected ratio ≥0.6 (40).

### Sharp and broad promoters

Promoters can be characterized as either sharp type or broad type, depending on whether they contain one dominant TSS or multiple TSSs (41). Based on the 188 FANTOM5 tissue libraries, we computed the dispersion index of CAGE tags for all promoter sequences, a metric that is conceptually similar to the standard deviation of tag counts (42). A low dispersion index indicates a sharp distribution of tags (or a dominant TSS), and a high dispersion index indicates a broad distribution of tags (or multiple TSSs). To compute dispersion indices, we counted tags between positions −50 and +50 relative to and on the same strand as the annotated TSSs for each library. Let *s*_*i*_ be the dispersion index for library *i* and *x*_*i*, *j*_ be the number of tags at position *j* relative to the annotated TSS in that library. Then let

$$\begin{equation*} s_i = \sqrt{\frac{1}{c_i}\sum _{j=-50}^{50}{(j-m_i)^2 x_{i,j}}}\textrm {,} \end{equation*}$$

where

$$\begin{equation*} c_i = \sum _{j=-50}^{50}{x_{i,j}},\qquad m_i = \frac{1}{c_i}\sum _{j=-50}^{50}{j x_{i,j}}\textrm {.} \end{equation*}$$

Promoters where the average dispersion index across libraries was ≤2.5 were considered sharp type, and broad type otherwise.

### Length analysis of bidirectionally transcribed enhancers

We extracted the length of bidirectionally transcribed enhancers from the FANTOM5 file (F5.hg38.enhancers.bed). Enhancers were classified into two groups depending on whether a CGI overlapped at least one of the two TSSs. Non-parametric analysis was performed with a Mann–Whitney *U* test.

### Quantifying tissue specificity

Genes are often classified as tissue specific or housekeeping depending on whether a large proportion of their expression is observed in one or a few tissues, or whether it is dispersed across all or most tissues. There are many methods to define this mathematically. A widely used and robust definition of tissue specificity is τ (tau), which ranges between 0.0 for housekeeping genes and 1.0 for tissue-specific genes (43,44). Let *x*_*i*_ be the expression of a gene in tissue *i* and *n* is the total number of tissues. Then

$$\begin{equation*} \tau = \frac{\sum _{i=1}^{n} 1 - \hat{x}_i}{n-1}\textrm {,} \end{equation*}$$

where

$$\begin{equation*} \hat{x}_i = \frac{x_i}{\max _{j\in [1,n]}\; x_j}\textrm {.} \end{equation*}$$

To compute τ in this study, expression per CAGE library was normalized and converted to expression per 29 distinct tissues and 36 distinct primary cells. For each promoter and tissue/primary cell, we then added up expression between positions −100 and +100 relative to and on the same strand as the TSS, scaled the result by a factor 1000, took the binary logarithm, and computed τ separately for the top *n* = 15 tissues and top *n* = 15 primary cells by total log-transformed expression over all promoters.

### Directionality analysis of bidirectionally transcribed enhancers

Directionality was calculated using pooled data from all 1829 CAGE libraries by counting CAGE tags falling within −200 bp of the reported mid position of the enhancer on the reverse strand (*R*) and within +200 bp of the mid position on the forward strand (*F*). Directionality is defined as (*F* − *R*)/(*F* + *R*), with a value close to 0.0 indicating balanced bidirectional transcription and a value close to −1.0 or 1.0 indicating unidirectional transcription (8).

### Statistical significance of local overrepresentation

**Figure 1.**
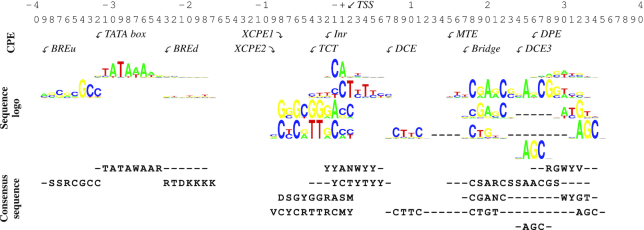
CPEs show localized overrepresentation with respect to the TSS and can be represented by PWMs. The sequence logo representing the PWM as well as the IUPAC consensus sequence with the most frequent nucleotides are shown (details in Supplementary Table S1).

To determine whether a CPE showed local overrepresentation, we partitioned the promoter sequences into two sets: The set CPE_+_ contained promoters in which the CPE is present at the expected location or up to two nucleotides upstream or downstream of the expected location (functional window) (Figure 1, Supplementary Table S2). CPE_−_ contained the remaining sequences. *P*-values for the cardinality *n* = |CPE_+_| were computed using the Gaussian and binomial distributions. To determine the standard score and expected occurrence probability, a 5-nucleotide window was shifted in 1 bp intervals across promoter sequences from position [−500, −495) relative to the TSS to (+195, +200]. Per location, we recorded the number of promoters where the start position of the CPE appeared inside the window and then used the average and standard deviation over all locations that did not overlap with the CPE’s functional window.

### CPE co-occurrence analysis

Fisher’s exact test was used to assess the statistical significance of co-occurrence of pairs of CPEs in promoters and enhancers. To carry out the test, we partitioned promoter sequences twice into two sets for each pair of distinct CPEs. CPE1_+_ (CPE2_+_) contained promoters in which the first (second) CPE was present in its functional window. Correspondingly, CPE1_−_ (CPE2_−_) contained promoters in which the first (second) CPE was not present in its functional window.

We then computed *P*-values for the overrepresentation of promoters showing co-occurrence of both CPEs

$$\begin{equation*} p = \sum _{i=0}^{\min \lbrace N-a,b,c,N-d\rbrace } \binom{a+b}{a+i}\binom{c+d}{c-i} \div \binom{N}{a+c}, \end{equation*}$$

as well as for overrepresentation of promoters lacking the first CPE but displaying the second

$$\begin{equation*} p = \sum _{i=0}^{\min \lbrace a,N-b,N-c,d\rbrace } \binom{c+d}{c+i}\binom{a+b}{a-i} \div \binom{N}{a+c}, \end{equation*}$$

where *N* is the count of promoters and

$$\begin{equation*} a=|\textrm {CPE1}_+ \cap \textrm {CPE2}_+|,\qquad b=|\textrm {CPE1}_+ \cap \textrm {CPE2}_-|\textrm {,} \end{equation*}$$

$$\begin{equation*} c=|\textrm {CPE1}_- \cap \textrm {CPE2}_+|,\qquad d=|\textrm {CPE1}_- \cap \textrm {CPE2}_-|\textrm {.} \end{equation*}$$

A Bonferroni correction was applied based on the total of 12 × 11/2 = 66 tests performed, corresponding to α = 0.05/66 = 7.58 × 10^−4^.

### H3K27ac analysis

BED files representing the results of H3K27ac ChIP-seq analysis were downloaded from the ENCODE data portal (45). The BED files were in narrowPeak format. We recorded whether the H3K27ac peaks in these files overlapped with a promoter or enhancer as defined above, and if so what the maximum H3K27ac signal was. We analyzed the files ENCFF757CYP, ENCFF779WYN, ENCFF698NII, ENCFF459UTL, ENCFF874YBQ, ENCFF196AMI, ENCFF587KQG, ENCFF812JNL, ENCFF110UVX, ENCFF783DOC, ENCFF168FUG, ENCFF088CLP and ENCFF626ZXA, representing the human cell types: hepatocyte, neural progenitor cell, trophoblast cell, mesendoderm cell, neural stem progenitor cell, mesenchymal cell, endodermal cell, mesodermal cell, ectodermal cell, bipolar neuron, neuroepithelial stem cell, neural cell and myotube originated from skeletal muscle myoblast.

### Density of ChIP-seq binding events

We used a dataset comprised of statistically significant ChIP-seq peaks for 599 human transcription factors (46). For our experiments, we restricted the analysis to the most reliable peaks (group A in hg38_cismotifs), which contain overlapping peaks detected in two or more experimental datasets and by at least two peak-calling tools, corresponding to a total of 124 unique transcription factors.

The promoter region of protein-coding genes was defined as comprising 500 nt upstream and 200 nt downstream of the TSS.

## RESULTS

In this work, we analyzed bidirectionally transcribed enhancers from the FANTOM5 project (36). 3587 of the 63 285 transcribed enhancers were associated with a CGI (5.7%), 59 698 enhancers (94.3%) were not. For some of the analyses, we compared the enhancers to a set of 29 598 promoter sequences, 17 336 of which were associated with a CGI (58.6%) and 12 262 of which were not (41.4%).

### CPEs show significant localized overrepresentation in transcribed enhancers

CPEs can be defined computationally based on overrepresentation of a sequence motif in a specified location with respect to the TSS (Materials and Methods). We reasoned that transcribed enhancers might demonstrate a comparable overrepresentation of CPEs because these enhancers are transcribed by RNAPII. In addition to the classic CPEs (TATA, BREu, Inr, DPE), we investigated eight other CPEs that have been proposed in the literature (Figure 1, Supplementary Tables S1 and S2).

**Figure 2.**
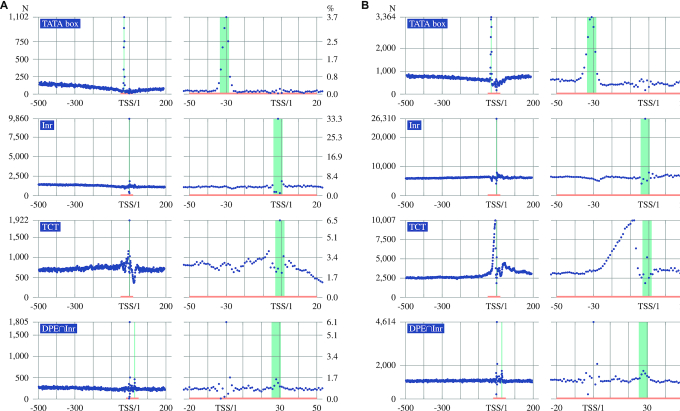
Local overrepresentation of CPEs in promoters and transcribed enhancers. (**A**) Promoters. (**B**) Transcribed enhancers. The panels show the occurrence counts for the CPEs TATA, Inr, TCT and DPE in positions −500 to +200 with respect to the TSS. DPE∩Inr: DPE was only called in sequences that contained an Inr motif. Each CPE showed a statistically significant overrepresentation in the region marked in green.

Of the twelve CPEs tested, eight displayed statistically significant local overrepresentation in core promoters of protein-coding genes (Supplementary Figure S1, Table S3). Seven of the CPEs demonstrated significant local overrepresentation with respect to the TSSs of the bidirectionally transcribed enhancer set (Supplementary Figure S2, Table S4). TATA, Inr and DPE showed clear peaks at the expected locations in both the promoter and the enhancer datasets. The DPE functions in coordination with Inr (47), and thus we only called DPE in sequences in which an Inr was present (Figure 2). The findings extend previous work that identified Inr, TATA, and BRE motifs associated with transcribed enhancers (9) by determining statistical significance and investigating eight additional CPEs. Interestingly, TCT appeared to have a higher degree of overrepresentation in the enhancers than in the promoters. In the enhancers, there was an apparent overrepresentation between positions −25 to −10, which is outside of the range given in the literature for TCT in promoters (48). It is unclear whether this observation points to a distinct biological role of TCT in enhancers.

### Correlation of occurrences of pairs of CPEs

Certain pairs of CPEs such as DPE/Inr have been shown to function cooperatively in some promoters (49). It was previously reported that several CPEs display statistically significant co-occurrence patterns; we confirmed previous reports of increased co-occurrence of DPE and Inr and reduced co-occurrence of TATA and BREu in the promoter dataset (26). The enhancer dataset also showed that DPE and Inr co-occurred significantly more often than chance and that TATA and BREu co-occurred significantly less often than expected by chance. Additionally, TATA box co-occurred with Inr significantly less and Inr co-occurred with BREu significantly more than expected by chance (Supplementary Tables S5 and S6).

The general transcription factor IID (TFIID) binds cooperatively to the Inr and DPE motifs (50). The observation provides a plausible explanation for the observed co-occurrence of these two CPEs. The reasons for the reduced co-occurrence of TATA and BREu remain unclear. It was shown in *Drosophila* that BREu suppresses the ability of the transcription factor Caudal to activate TATA-dependent promoters, indicating that BREu contributes to CPE-mediated transcriptional regulation in TATA-containing promoters (51). Speculatively, similar interactions in human could be responsible for the observed anticorrelation. To our knowledge, no experimental evidence exists for this or for the other correlations we observed.

### Dispersed transcription initiation associated with CGIs in enhancers

CAGE tag analysis of promoters showed that CGI-associated promoters tend to initiate transcription from a broad region, while non-CGI-associated promoters tend to have sharp peaks of transcription initiation (41). We confirmed previous findings that the majority of CGI promoters are broad, while the majority of non-CGI promoters are sharp. In contrast to promoters, most enhancers have a sharp peak of transcription initiation. However, as with promoters, the proportion of CGI enhancers is substantially higher in the broad group compared to the sharp group (Table 1).

**Table 1.** Association of CGIs with transcription initiation patterns

	Promoters			Enhancers		
Type	Overall	CGI	Non-CGI	Overall	CGI	Non-CGI
Sharp	2932	525	2407*	91661	2307	89354*
	(9.9%)	(17.9%)	(82.1%)	(72.4%)	(2.5%)	(97.5%)
Broad	26321	16734*	9587	28551	2297*	26254
	(88.9%)	(63.6%)	(36.4%)	(22.6%)	(8.0%)	(92.0%)

Counts of sharp-type and broad-type promoter and enhancer sequences. 345 (1.2%) of 29 598 promoter transcripts and 6358 (5.0%) of 126 570 enhancer transcripts were not classified because of insufficient CAGE tag coverage. *P-values <1.2 × 10^−38^ computed with Fisher’s exact test.

### CGI-associated transcribed enhancers are longer than other transcribed enhancers

**Figure 3.**
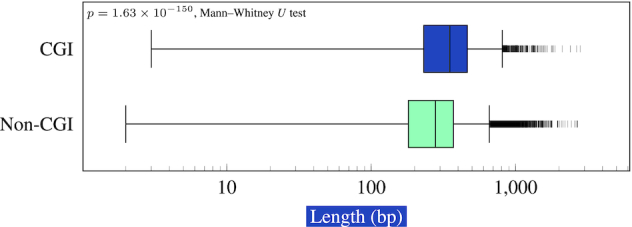
Length distribution of transcribed enhancers. The mean length of CGI-associated enhancers was 384.0 bp, that of non-CGI-associated enhancers was 289.2 bp.

We compared the lengths of the bidirectionally transcribed enhancers according to whether an enhancer overlaps with a CGI on one or both of its TSSs (present) or not (absent). While the overall length distribution was similar (Figure 3), the mean length was significantly higher for the CGI-present group (384.0 bp versus 289.2 bp in the absent group; *P* = 1.63 × 10^−150^ by the Mann–Whitney *U* test). 93 CGI-present enhancers were over 1000 bp in length (2.59% of a total of 3587), while only 269 CGI-absent enhancers were over 1000 bp (0.45% of a total of 59,698).

### Tissue specificity and expression is associated with TATA box and CGI presence

**Figure 4.**
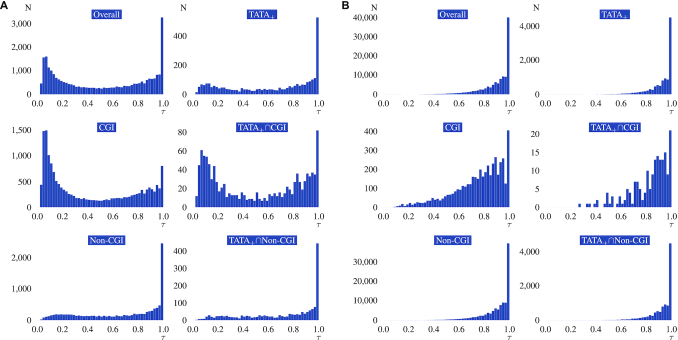
Association of CGI and TATA presence with tissue specificity. (**A**) Promoters. Distribution of τ across all promoters (upper left panel) and across all promoters with a TATA box (upper right). The second and third rows show the distribution for promoters with CGIs and without CGIs. (**B**) Transcribed enhancers. The meaning of the individual panels is the same as in part A but for enhancer sequences.

The molecular mechanisms controlling tissue specificity remain incompletely understood, but measures including Shannon entropy and τ (tau) have been used to characterize the overall tissue specificity of a gene. These measures characterize the extent to which a gene tends to be tissue-specific or broadly expressed (housekeeping), irrespective of the specific tissue or tissue in which it is expressed (44). Some features of promoters have been associated with tissue-specificity, including the presence of a TATA box and the lack of a CGI (29). We therefore compared the distributions of τ, a measure of tissue specificity that varies from 0 (completely ubiquitous) to 1 (completely specific) in promoters and enhancers (Figure 4). As expected, the τ values indicated significantly higher tissue-specificity for promoters lacking CGIs both in tissues and primary cells (Table 2 shows results for tissues and Supplementary Table S7 shows results for primary cells; Supplementary Tables S8 and S9 show analogous results for all twelve CPEs investigated in this study). An analogous significant difference was noted for enhancers, again both in tissues and primary cells.

**Table 2.** Association of CGI and TATA presence with tissue specificity (τ)

**A**	Promoters			Enhancers		
	CGI	Non-CGI	*P*-value	CGI	Non-CGI	*P*-value
Overall	0.278	0.771	<10^−300^	0.795	0.944	<10^−300^
**B**	Promoters			Enhancers		
	TATA_+_	TATA_−_	*P*-value	TATA_+_	TATA_−_	*P*-value
Overall	0.726	0.480	1.9 × 10^−77^	0.961	0.939	1.1 × 10^−131^
CGI	0.482	0.269	1.7 × 10^−22^	0.878	0.791	7.3 × 10^−8^
Non-CGI	0.820	0.761	1.7 × 10^−9^	0.962	0.942	3.6 × 10^−108^

The table shows τ medians of different subsets of promoters and enhancers. (**A**) τ medians according to CGI status. (**B**) τ medians for CGI and non-CGI promoters and enhancers according to the presence (TATA_+_) or absence (TATA_−_) of a TATA box. *P*-values were calculated with the Mann–Whitney *U* test. The values are derived from tissue data.

In accordance with previous findings (8), we found that the enhancers showed a much higher degree of tissue specificity than promoters. Within the set of all enhancers, non-CGI-associated enhancers showed a significantly higher degree of specificity in both tissue and primary cell samples. The presence of a TATA box was associated with a significantly higher degree of specificity in both sample types. This finding was statistically significant over the entire set of enhancers and all comparisons were significant in the subsets of CGI and non-CGI enhancers.

### Directionality, expression, and H3K27ac density of transcribed enhancers is associated with presence of CGIs

**Figure 5.**
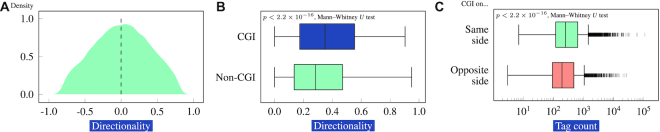
Directionality of transcribed enhancers is associated with CGIs. (**A**) Distribution of the directionality of 63 285 transcribed enhancers. (**B**) The absolute value of the directionality is significantly higher for enhancers that overlap with a CGI than for those that do not. (**C**) The overall CAGE tag count of enhancers associated with CGIs is higher for transcription in the direction of the CGI (same side) than on the opposite side of the enhancer.

CGIs colocalize with the promoters of constitutively expressed genes and ∼40% of genes with a tissue-restricted expression profile (52). Therefore, one would expect that CGI-associated promoters would be found to have more CAGE tags than non-CGI-associated promoters across the FANTOM5 atlas. Indeed, CGI-associated promoters had a median of 59 851 total CAGE tags, while CGI-free promoters had a median of only 3287 (*P* < 10^−300^, Mann–Whitney *U* test; Supplementary Figures S3 and S4). We therefore investigated whether there is a relationship between the presence of a CGI overlapping one of the TSSs of a transcribed enhancer with the directionality of transcription. Indeed there was a statistically significant increase in both directionality and total number of tags for CGI-associated enhancers (Figure 5).

**Figure 6.**
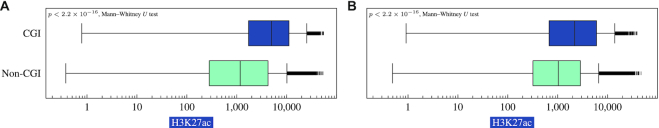
Distribution of ChIP-seq H3K27ac signal. (**A**) Promoters. The distribution of maximum H3K27ac signal among promoters overlapping H3K27ac peaks. The mean was significantly different (7683 versus 3662). (**B**) Enhancers. The mean was significantly different (4549 versus 2365). Data are shown on a log_10_ scale.

H3K27ac is a histone mark that can be associated with active promoters and enhancers (13,53). 89.8% of the FANTOM5 enhancers overlap with H3K27ac signal in at least one experiment included in the Ensembl regulatory build, compared to 67.8% of length-matched control sequences (Supplementary Table S10). We then analyzed the promoter and enhancer datasets with respect to H3K27ac peaks from 13 narrowPeak BED files from the ENCODE project (Materials and Methods, Figure 6). There was a significantly higher maximum H3K27ac signal in the CGI promoters and CGI enhancers than in their non-CGI counterparts (Figure 6).

### Global transcription factor binding is more frequent in enhancer-associated CGIs than in CGI-free enhancers

CGIs tend to lack sequence conservation over long evolutionary distances, and it is thought that their GC richness may increase the probability of binding of ubiquitous transcription factors such as SP1 (54). DNA footprinting experiments suggest that protein DNA interactions at CGI-overlapping promoters are concentrated between the 5’ region of the CGI and the TSS site of the promoter (54). We therefore asked whether there is an enrichment of TFBSs in the vicinity of promoter- and enhancer-associated CGIs. We are not aware of a genome wide footprinting dataset across multiple tissues that would allow a detailed comparison with the FANTOM5 CAGE dataset. Therefore, we chose to analyze a comprehensive compendium of data accumulated from published human transcription factor ChIP-seq experiments (46). The average length of the ChIP-seq peaks was 491.5 ± 222.4 nt. It is not unambiguously possible to assign the exact location of protein binding within a ChIP-seq peak, and so this relatively low resolution is a limitation of our analysis.

As hypothesized on the basis of the above mentioned footprinting results, there was a significantly higher rate of ChIP-seq binding events at promoter-associated CGIs (5.06 per 1000 nt) as compared to promoters not associated with CGIs (1.89 per 1000 nt). For the enhancers, the density of ChIP-seq binding events at enhancer-associated CGIs was 2.46 per 1000 nt compared to 1.15 per 1000 nt for enhancers not associated with CGIs (*P* < 10^−300^, Mann–Whitney *U* test). If we instead compare the body of enhancers, there were 3.26 ChIP-seq binding events for enhancers associated with a CGI, again compared to 1.15 per 1000 nt for enhancers not associated with CGIs (*P* < 10^−300^, Mann–Whitney *U* test). There was also a total higher number of ChIP-seq binding events at enhancers associated with CGIs (Supplementary Figure S5). Therefore, CGI-associated promoters and enhancers both displayed higher rates of transcription factor ChIP-seq peaks than promoters and enhancers not associated with CGIs.

**Figure 7.**
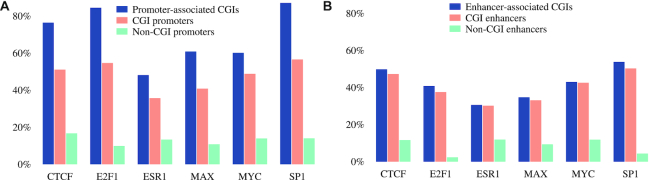
Cistrome-wide transcription factor ChIP-seq peaks. Binding of most transcription factors was substantially more frequent in CGI-associated promoters or enhancers than in the promoters or enhancers without CGIs. Binding was the most frequent within the CGI sequences themselves (which tend to partially overlap with promoter/enhancer sequences). (**A**) Promoters. 17 336 promoters were associated with one or more CGIs. 12 262 were not associated with a CGI. (**B**) Transcribed enhancers. 3587 enhancers were associated with one or more CGIs. 59 698 were not associated with a CGI.

Figure 7 shows a comparison between promoters and transcribed enhancers with the six most commonly encountered transcription factors. In all six cases, the frequency of ChIP-seq peaks was significantly higher in enhancers or promoters associated with a CGI or within the CGI itself (see Supplementary Tables S11 and S12 for more transcription factors). 1787 (49.8%) of the enhancer-associated CGIs contained a CTCF site, and 1931 (53.8%) contained an SP1 site. In contrast, only 2616 non-CGI-associated enhancers had an SP1 site (4.4%) and only 6959 had a CTCF site (11.7%). Ubiquitously active CGI promoters are enriched for transcription factor binding motifs (TFBMs) for factors including SP1 and E2F (55). MYC is an oncoprotein that binds DNA as an obligatory heterodimer with MAX that has a high affinity for a CpG-containing palindromic E-box sequence CACGTG. MYC has a known tendency to colocalize with promoter-associated CGIs (56). Genes with GC-rich promoter sequences can be regulated through the interaction of estrogen receptors with SP1 (57). The fact that the most frequently binding transcription factors display CGI-dependent enrichment in both promoters and transcribed enhancers suggests the possibility that they may play a similar role for promoters and enhancers.

### Genes regulated by CGI-associated enhancers are enriched in functions related to transcriptional regulation

The FANTOM5 consortium linked enhancer usage to the expression of genes by correlating counts of CAGE tags for enhancers and genes across multiple CAGE libraries. Correlation between the expression profile of an enhancer and the TSS of a gene can be interpreted as suggestive evidence that the gene is regulated by the enhancer, an interpretation that was supported by an analysis of ENCODE ChIA-PET data by the FANTOM5 authors. Each RefSeq TSS was associated with a mean of 4.9 FANTOM5 enhancers, and each of these enhancers was associated with a mean of 2.4 TSSs (8).

Out of a total of 13 881 genes that were putatively regulated by at least one enhancer in that dataset, 2743 were regulated by CGI-associated enhancers. Seventeen Gene Ontology (GO) terms were significantly overrepresented with a *P*-value <0.001 (Supplementary Table S13). Among the most significant terms were transcription regulator activity (GO:0140110), chromosome (GO:0005694), and regulatory region nucleic acid binding (GO:0001067), indicating that many of the genes likely to be regulated by CGI-associated enhancers are themselves involved in transcriptional regulation or encode gene products that have a chromosomal location.

## DISCUSSION

A major goal of biology is to understand the molecular mechanisms that mediate tissue- and developmental stage-specific gene regulation patterns that underlie development, cell identity and function, and whose malfunction contributes to disease. The last decade has seen a paradigm shift in our understanding of enhancers, which were traditionally thought to be strictly distinct from promoters. As noted in the introduction, many enhancers are bidirectionally transcribed and in many cases their function depends on the transcription products (eRNAs) or perhaps on the transcription process itself. In the last decade, numerous studies have shown that enhancers and promoters share many features, including similar sequence motifs, transcription machinery, chromatin environment, and changes in activity upon binding of activators or repressors (58). However, the available data do not unambiguously allow one to determine whether enhancer function is mediated by binding proteins interacting with the transcription machinery, by the transcribed eRNAs, by the transcription process itself, or a combination of these.

The contribution of the current study centers around a detailed analysis of the relation between enhancer and promoter characteristics and the presence or absence of CGIs. The function of CGIs is not completely understood but is thought to involve the establishment of transcriptionally permissive chromatin states by destabilizing nucleosomes and attracting DNA-binding proteins (52,54). CGIs have been associated with numerous biological processes including early embryonic development (39). Our study shows that even though there are substantial differences in the proportion of CGI-associated promoters and enhancers (over half of promoters and only roughly 6% of enhancers), these sets of elements display consistent associations of numerous sequence properties and functional characteristics with the presence or absence of CGIs.

### Enhancer detection methods

Enhancers are defined as DNA sequences that modulate the expression of target genes in a space and time-dependent manner, whereby the relative orientation of the enhancer to the target genes is irrelevant and the enhancers can be located kilobases or even megabases distant from their target promoters. This does not easily lead to an operational definition of an enhancer that can be used for a specific and sensitive enhancer assay. Correspondingly, we still do not have a comprehensive and accurate catalog of mammalian enhancers. The classic and still generally accepted definition of an enhancer focuses on the functional capacity of DNA to enhance transcription of a reporter gene in an orientation and position-independent manner (59,60). The enhancer assays introduce a candidate enhancer sequence upstream of a minimal promoter that can activate transcription of a reporter gene whose expression levels can be quantified by LacZ staining, luciferase assays, or other methods. Recently, several massively parallel reporter assays (MPRAs) have been introduced that test candidate fragments in parallel using next-generation sequencing (NGS) technologies. For instance, with self-transcribing active regulatory region sequencing (STARR-seq), a reporter library is cloned and reporter transcripts are counted by NGS. The reporter library can be assembled from DNA fragments enriched for regions of interest such as open chromatin (ATAC-seq) or TFBSs (ChIP-seq) (61). By definition, sequences identified by STARR-seq satisfy the classic definition of enhancer sequences mentioned above, although the results of the method can be confounded by systematic sources of bias (62). Additional methods include analysis of local enrichment of histone modifications such as H3K27ac and H3K4me1 (see above), increased chromatin accessibility (63), as well as the CAGE assays for enhancer transcription that have been discussed in this work.

The enhancer candidates defined by these methods often do not show a high degree of overlap, even if one only considers methods with a functional readout such as STARR-seq, CAGE-tag analysis, and reporter gene assays of target enhancer sequences. This could be due to technical limitations of the assays, differences in the biological systems being analyzed, or other factors. Table 3 shows that the overlap between enhancer candidates from three MPRA studies with FANTOM5 enhancers is generally <10%. The overlap of the CGI-associated enhancers with the STARR-seq peaks ranged from 5.1 to 11.4%, and the non-CGI-associated enhancers showed an overlap of 3.5–9.6%. While further work will be required to understand whether all FANTOM5 enhancers would show activity in STARR-seq assays if done in appropriate cell types, we conclude from this evidence that the degree of overlap of CGI enhancers and non-CGI enhancers is of the same order of magnitude.

**Table 3.** Comparison of FANTOM5 and MPRA enhancer sets

		Enhancer coverage	
	Count	CGI	Non-CGI
HeLa-S3 set, shortlisted regions (62)	71930	183 (5.1%)	5723 (9.6%)
GM12878 set, active regions (64)	66214	410 (11.4%)	4948 (8.3%)
Human ESC set, active regions (60)	32223	369 (10.3%)	2080 (3.5%)

Enhancer coverage lists the number and percentage of FANTOM5 enhancers overlapping regions in the three MPRA sets. MPRA sets were converted to hg38 using liftOver.

The FANTOM5 dataset is unique in that it allows the precise boundaries of enhancers to be defined, which is a prerequisite for some of the analysis approaches presented here such as the localized overrepresentation of CPEs.

### CPEs

Transcription initiation at promoters requires the stepwise assembly of GTFs (TFIID, TFIIA, TFIIB, TFIIF, TFIIE, TFIIH) and RNAPII. The TATA-binding protein (TBP) subunit of TFIID can bind the TATA box found in some core promoters, and other subunits of TFIID (the TBP-associated factors or TAFs) appear to interact with Inr and DPEs (65). However, the binding partners of other CPEs, if any, have not been definitively elucidated. GTFs bind not only to promoters but also to transcribed enhancers (66).

CPEs such as the TATA box are computationally defined by overrepresentation of a sequence motif in a specific location with respect to the TSS of a promoter (26). The presence of CPEs has been noted in the transcribed enhancers previously in humans (8) and *Drosophila* (67), but to the best of our knowledge, we have shown for the first time that there is a statistically significant overrepresentation of CPEs in transcribed enhancer sequences. Additionally, we have shown that a comparable ‘synergy’ (correlation of occurrences of some pairs of CPEs) exists for enhancers as has been shown previously for promoters (26).

The TATA box has previously been associated with overall tissue specificity of gene expression (29). We show here that it is also associated with the overall (predicted) tissue specificity of enhancers, albeit to a lower extent. The effect is related to but not entirely explained by the anticorrelation of TATA boxes with CGIs. The fact that the presence of TATA box is correlated with tissue specificity in both promoters and enhancers suggests that TATA may play a similar role in both promoters and enhancers.

Our findings of similarities in the distribution of CPEs in promoters and transcribed enhancers provides additional support for a similar biological role of GTFs in both classes of genomic element.

### CGI-dependent characteristics of transcribed enhancers

Our results have demonstrated that CGI-enhancer associated transcripts are longer, have a lower degree of tissue specificity (τ), and a higher overall expression than enhancers lacking a CGI, which is comparable in direction if not in amplitude to the analogous findings in promoters. Our finding of higher overall expression in CGI-associated enhancers may be related to a recent finding that GC dinucleotide repeat motifs are enriched in broadly active enhancers compared to both the genomic background and context-specific enhancers (34).

### Transcription-factor binding

Chromatin immunoprecipitation coupled with next-generation sequencing (ChIP-seq) is a powerful technology to identify the genome-wide locations of transcription factors and other DNA-binding proteins. ChIP-seq can identify both sharp peaks typically associated with sequence-specific transcription factors, as well as broad histone-modification signals, and involves formaldehyde-mediated cross-linking of chromatin followed by fragmentation of protein-DNA complexes into short fragments, which are then subjected to immunoprecipitation using an antibody directed against a protein of interest (68,69). We leveraged a database of ChIP-seq peaks that was derived from published studies, and analyzed high-quality data derived from 124 transcription factors. We observed a range of peak frequencies across promoters and enhancers. The six most frequently observed transcription factors are known to favor GC rich sequences. Although the overall frequency of binding is lower than for promoters, the factors display a highly significantly increased binding in enhancers associated with CGIs or the associated CGI sequences. This finding is analogous to the comparable finding in promoters. We interpret the finding as suggesting that CGIs play a similar role as in promoters for the subset of enhancers that are associated with them, namely by promoting binding of transcription factors, including especially factors that bind to GC-rich sequences.

## CONCLUSION

In this work, we have investigated associations of a number of characteristics of transcribed enhancers and their relations with the presence or absence of CGIs. Although transcribed enhancers are likely to represent a heterogeneous set of genomic elements with different regulatory mechanisms, we have shown that the subset of CGI-associated enhancers display a number of distinguishing characteristics that differentiate them from non-CGI-associated enhancers. CGI-associated enhancers are longer, display a higher degree of directionality and strength of expression, show a higher frequency of transcription factor binding events, more H3K27ac signal, and putatively regulate a set of genes enriched for functions including transcriptional regulation.

## Supplementary Material

gkaa223_Supplemental_File
