# Supplementary material for: Pervasive and CpG-dependent promoter-like characteristics of transcribed enhancers
Source: Nucleic Acids Res. 2020 Apr 27;48(10):5306–17. doi: 10.1093/nar/gkaa223 (PMC7261191; doi:10.1093/nar/gkaa223)
Supplement: gkaa223_Supplemental_File [file gkaa223_supplemental_file.pdf]

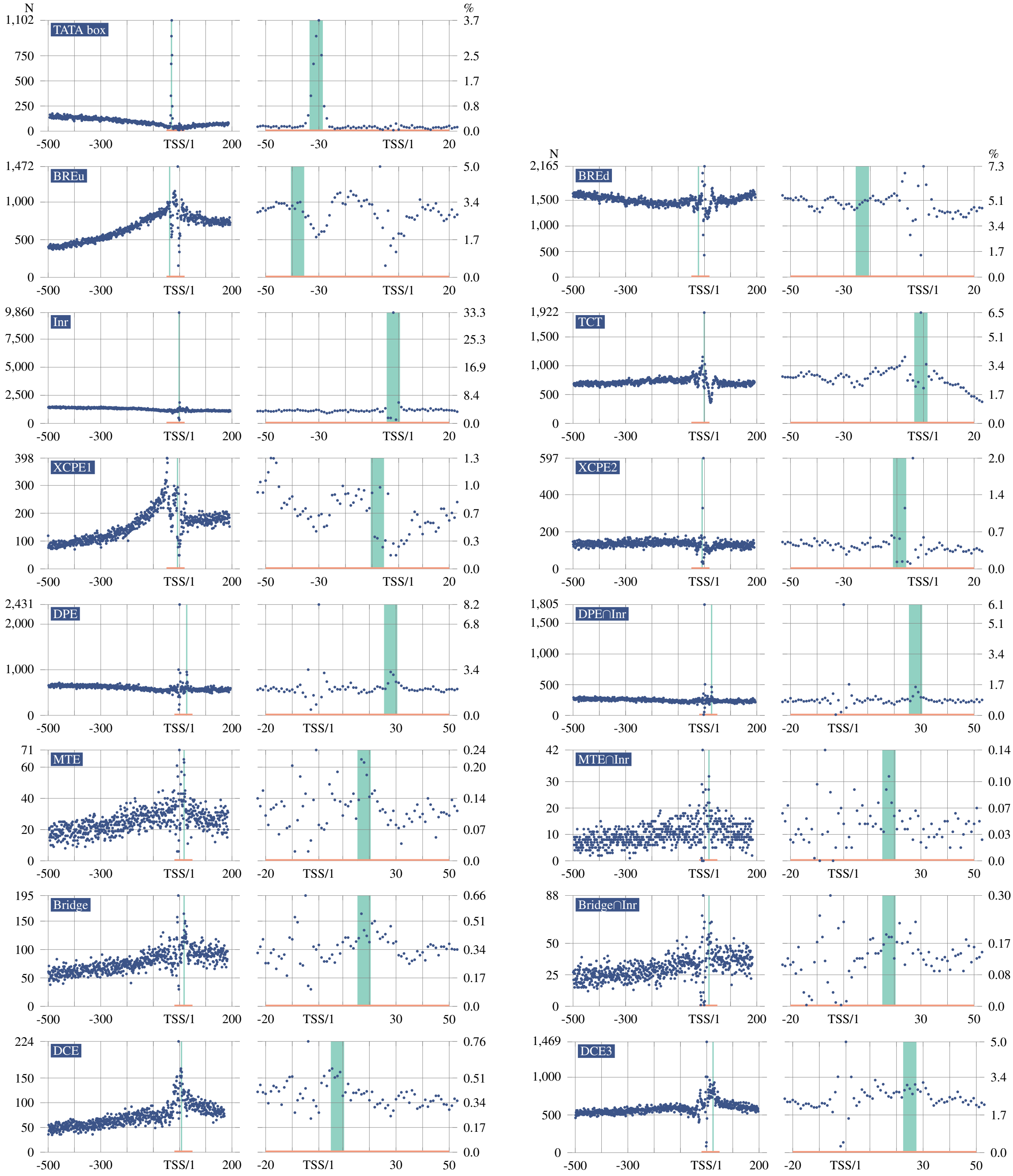

Figure S1 Localized overrepresentation of core promoter elements (CPEs) in promoter sequences Counts of each CPE are shown as a function of position with respect to the transcription start site (TSS) across 29,598 promoters derived from the Eukaryotic Promoter Database New (EPDnew) dataset, version 006 (hg38) [1]. DPE∩Inr: DPE was only called in sequences that contained an Inr motif (analogously for MTE∩Inr and Bridge∩Inr). The regions marked in green indicate the location of expected occurrence of CPEs.

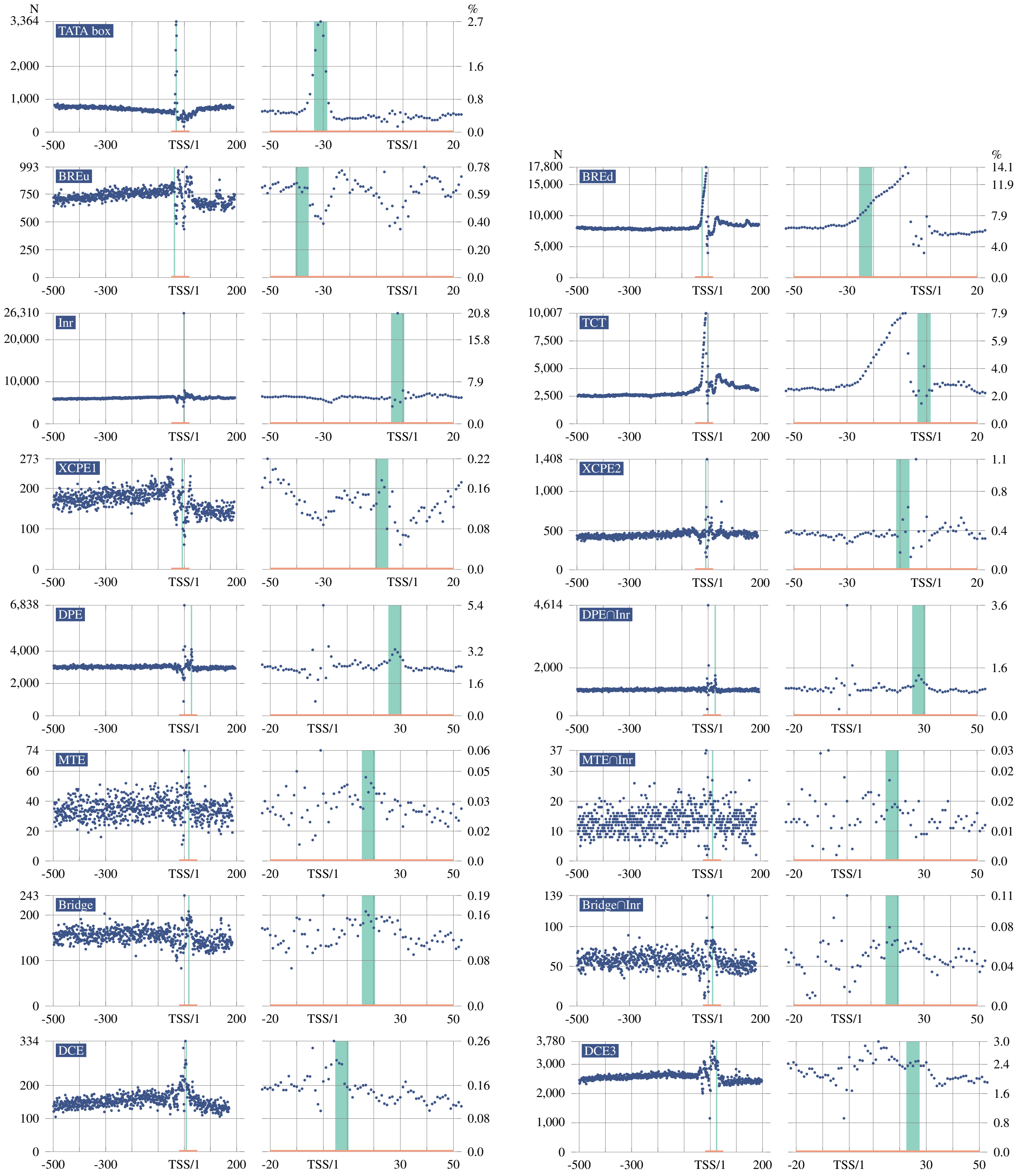

Figure S2 Localized overrepresentation of CPEs in enhancer sequences
Counts of each CPE are shown as a function of position with respect to the TSS (separately for each strand) across 63,285 transcribed enhancers from the FANTOM5 dataset (hg38) [2]. DPE∩Inr: DPE was only called in sequences that contained an Inr motif (analogously for MTE∩Inr and Bridge∩Inr). The regions marked in green indicate the location of expected occurrence of CPEs. As with TCT (see main manuscript), there was an apparent overrepresentation of BREd outside of the expected range.

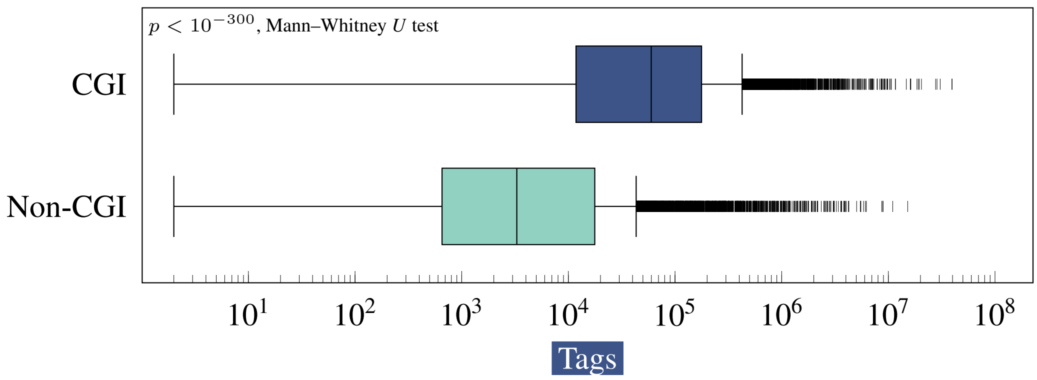

Figure S3 CAGE tag counts for CpG island (CGI) and non-CGI promoters
There was a mean of 216,208 CAGE tags for CGI-associated promoters, and a mean of 55,573 tags for non-CGI-associated promoters (medians: CGI-associated 59,851, non-CGI-associated 3287).

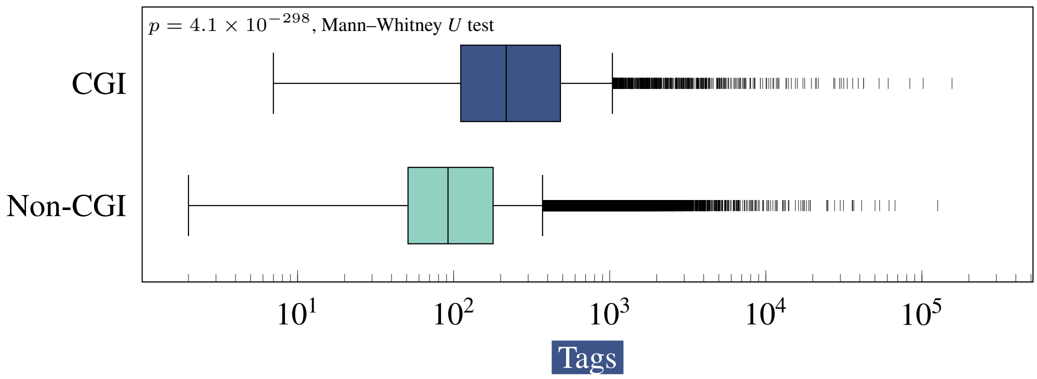

Figure S4 CAGE tag counts for CGI and non-CGI enhancers
There was a mean of 781 tags for CGI-associated enhancers, and a mean of 185 for non-CGI-associated enhancers (medians: CGI-associated 217, non-CGI-associated 93).

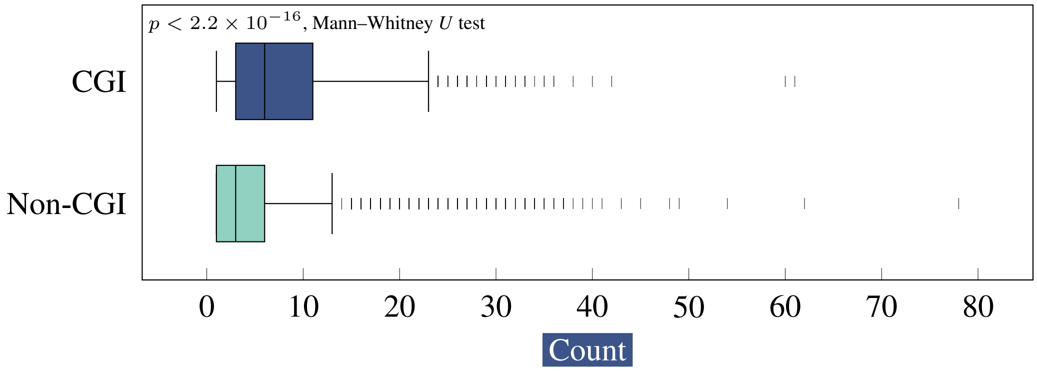

Figure S5 Total number of ChIP-seq peaks in CGI- and non-CGI-associated enhancers
Total number of ChIP-seq peaks for 124 unique transcription factors from group A (most reliable peaks in `hg38_cismotifs`) [3]. The analysis is analogous to the analysis in Figure 7 of the main manuscript, but total ChIP-seq peaks for all 124 transcription factors are analyzed.

CPE	PWM	PCM	Source
TATA box	A [-0.68{-3.02 0.00 -4.75 0.00 0.00 0.00 0.00 -0.12}-1.03 -0.58 -0.44 -0.42 -0.69 -0.56]<br>C [-0.06 -1.79 -5.60 -3.59 -5.44 -4.90 -4.24 -3.92 -1.31 -0.06 0.00 0.00 -0.06 -0.23 -0.25]<br>G [0.00 -2.93 -4.76 -4.90 -3.98 -4.90 -2.74 -1.48 0.00 0.00 -0.16 -0.01 0.00 0.00 0.00]<br>T [-1.74 0.00 -2.71 0.00 -2.56 -0.34 -3.69 -0.23 -1.51 -1.06 -1.50 -0.85 -0.69 -0.56 -0.64]	A [61{ 16 352 3 354 268 360 222 155} 56 83 82 82 68 77]<br>C [145 46 0 10 0 0 3 2 44 135 147 127 118 107 101]<br>G [152 18 2 2 5 0 20 44 157 150 128 128 128 139 140]<br>T [31 309 35 374 30 121 6 121 33 48 31 52 61 75 71]	[4]
BREu	A [0.00{-1.37 -2.62 -0.72 -1.42 -5.34 -5.19 -2.39}]<br>C [-0.55 -0.46 0.00 -1.19 0.00 -5.34 0.00 0.00]<br>G [-1.69 0.00 -0.81 0.00 -2.13 0.00 -2.94 -2.66]<br>T [-2.27 -4.63 -4.81 -4.66 -2.51 -5.34 -3.08 -2.00]	A [12{ 3 1 6 4 0 0 1}]<br>C [7 8 14 4 15 0 20 18]<br>G [2 11 7 12 2 22 1 1]<br>T [1 0 0 0 1 0 1 2]	[5]
BREd	A [{-0.54 -1.39 -0.21 -1.65 -1.22 -2.13 -0.83}]<br>C [-1.54 -0.84 -0.82 -1.01 -0.60 -1.12 -0.45]<br>G [0.00 -0.95 -0.18 -0.11 0.00 -0.37 -0.32]<br>T [-0.93 0.00 0.00 0.00 -0.12 0.00 0.00]	A [{10 5 10 3 4 2 6}]<br>C [4 8 6 6 8 6 9]<br>G [19 8 11 15 15 13 10]<br>T [8 20 14 17 14 20 16]	[6]
Inr	A [{-0.86 -5.56 0.00 -1.59 -0.47 -0.40 -0.70}-0.60]<br>C [-1.01 0.00 -5.33 -0.41 -0.26 0.00 -0.17 -0.03]<br>G [-0.15 -5.56 -5.33 0.00 -4.39 -0.79 -0.25 -0.52]<br>T [0.00 -5.56 -3.02 -0.32 0.00 -0.44 0.00 0.00]	A [{ 49 0 288 26 77 67 45} 50]<br>C [48 303 0 81 95 118 85 96]<br>G [69 0 0 116 0 46 73 56]<br>T [137 0 15 80 131 72 100 101]	[4]
TCT	A [-2.09 -3.00 -4.57{-4.63 -5.29 -5.13 -4.62 -3.28 -2.91 -2.20}-1.66]<br>C [0.00 -1.43 -0.83 0.00 0.00 -5.13 0.00 -2.32 -0.45 0.00 0.00]<br>G [-1.32 -0.84 -0.61 -4.63 -3.60 -3.52 -2.76 -5.08 -2.86 -1.53 -1.02]<br>T [-1.40 0.00 0.00 -0.44 -5.29 0.00 -0.03 0.00 0.00 -0.25 -1.38]	A [3 1 0{ 0 0 0 0 1 1 2} 4]<br>C [31 7 11 30 49 0 23 4 18 25 30]<br>G [8 12 11 0 1 1 1 0 1 4 9]<br>T [8 30 28 20 0 49 26 45 30 19 7]	[7]
XCPE1	A [{-5.11 -4.65 -5.27 -5.19 -5.08 -5.01 -1.81 0.00 -4.87 -2.08}]<br>C [-5.11 -0.60 -5.27 0.00 -5.08 -5.01 -5.01 -5.06 0.00 0.00]<br>G [0.00 0.00 0.00 -5.19 0.00 0.00 0.00 -5.06 -0.45 -5.04]<br>T [-5.11 -4.65 -5.27 -2.24 -5.08 -5.01 -5.01 -5.06 -4.87 -5.04]	A [{ 0 0 0 0 0 0 3 21 0 2}]<br>C [0 6 0 19 0 0 0 0 14 19]<br>G [21 15 21 0 21 21 18 0 7 0]<br>T [0 0 0 2 0 0 0 0 0 0]	[8]
XCPE2	A [{-0.59 -5.23 -4.40 -5.27 0.00 -5.33 -5.36 -1.23 -5.36 -0.14 -4.58}]<br>C [-0.16 0.00 -0.08 0.00 -4.97 -5.33 -5.36 -5.34 0.00 0.00 0.00]<br>G [0.00 -5.23 -4.40 -5.27 -0.36 -5.33 -5.36 0.00 -5.36 -4.62 -4.58]<br>T [-4.35 -5.23 0.00 -5.27 -4.97 0.00 0.00 -5.34 -5.36 -4.62 -0.01]	A [{13 0 0 0 26 0 0 14 0 20 0}]<br>C [23 52 26 52 0 0 0 0 52 32 29]<br>G [16 0 0 0 26 0 0 38 0 0 0]<br>T [0 0 26 0 0 52 52 0 0 0 23]	[9]
DPE	A [-0.28 0.00{ 0.00 -3.00 0.00 -1.82 -0.71}-0.84 -0.10]<br>C [-0.46 -0.38 -2.55 -0.26 -2.90 -0.42 -0.25 -0.47 -0.10]<br>G [0.00 -1.75 -0.35 0.00 -3.45 -2.23 0.00 -1.16 -0.23]<br>T [-1.10 -0.47 -1.31 -2.55 -0.31 0.00 -4.56 0.00 0.00]	A [24 36{41 2 43 7 16}15 19]<br>C [18 21 3 30 2 30 31 21 22]<br>G [30 6 28 47 1 4 35 11 17]<br>T [10 19 10 3 36 41 0 35 24]	[10]
MTE	A [-1.07 -1.39 -1.37{-5.24 -1.11 0.00 -0.54 -5.27 -4.59 -0.72 0.00 -0.32 -5.28 -5.11 -4.94}-0.18 -0.63 -1.54 -1.23]<br>C [-0.54 -0.43 -0.92 0.00 -4.98 -5.10 -4.93 0.00 -0.30 0.00 -5.21 -4.71 0.00 -5.11 -0.87 -4.57 0.00 -0.65 0.00]<br>G [-0.41 -4.66 -1.21 -5.24 0.00 -5.10 0.00 -5.27 0.00 -0.05 -5.21 0.00 -5.28 0.00 0.00 -4.57 -4.45 0.00 -0.61]<br>T [0.00 0.00 0.00 -5.24 -4.98 -5.10 -4.93 -5.27 -4.59 -4.44 -5.21 -1.45 -5.28 -5.11 -4.94 0.00 -0.17 -4.78 -0.11]	A [1 1 1{0 2 9 4 0 0 2 9 4 0 0 0}4 2 1 1]<br>C [2 3 2 9 0 0 0 9 4 3 0 0 9 0 2 0 4 3 3]<br>G [2 0 1 0 7 0 5 0 5 4 0 4 0 9 7 0 0 5 2]<br>T [4 5 5 0 0 0 0 0 0 0 0 1 0 0 0 5 3 0 3]	[11]
Bridge	A [-0.95{-1.82 -5.15 0.00 -1.95 -2.81 -0.68 -5- -1.14 0.00 -5.20 -5.44 -4.72}-1.61]<br>C [-0.19 0.00 -2.54 -4.87 -1.19 0.00 -1.31 0.00 -5.18 -1.14 -5.44 -1.47 0.00]<br>G [0.00 -1.91 0.00 -1.51 0.00 -5.26 0.00 -0.23 -5.18 -5.20 0.00 -0.98 -0.27]<br>T [-2.03 -4.90 -5.15 -4.87 -5.02 -5.26 -0.33 -0.14 -1.41 0.00 -5.44 0.00 -0.11]	A [3{ 2 0 14 2 1 3 -5- 2 13 0 0 0} 1]<br>C [7 13 1 0 4 16 2 5 0 5 0 2 6]<br>G [6 2 16 3 11 0 7 5 0 0 17 4 4]<br>T [1 0 0 0 0 0 5 5 4 12 0 11 6]	[12]*
DCE	A [-1.23{-2.98 -2.94 -2.55 -3.53 -0.22 -4- -1.12 -3.29 -2.53 -3.60 -1.49 -1.05 -8- -1.23 0.00 -5.52 -5.36}-0.12]<br>C [-1.29 0.00 -1.93 -1.72 0.00 0.00 -0.47 0.00 -2.90 -2.20 -0.92 -0.61 0.00 -5.19 -5.52 0.00 0.00]<br>G [0.00 -2.71 -2.27 -0.87 -2.41 -0.15 0.00 -3.42 -2.00 0.00 -1.56 0.00 -0.50 -5.19 0.00 -5.36 -0.09]<br>T [-1.03 -2.96 0.00 0.00 -2.70 -0.20 -1.26 -3.78 0.00 -3.02 0.00 -0.97 -1.27 -5.19 -5.52 -5.36 -0.27]	A [18{ 4 3 4 2 22 -4- 16 3 6 2 14 16 -8- 17 108 0 0}20]<br>C [19 93 12 12 93 34 34 100 5 11 21 28 43 0 0 108 36]<br>G [46 6 8 20 7 27 43 3 13 90 13 45 35 0 108 0 29]<br>T [25 5 85 72 6 25 15 2 84 5 60 19 13 0 0 0 23]	[13]
DCE3	A [-1.46{ 0.00 -5.52 -5.36}-0.12]<br>C [0.00 -5.19 -5.52 0.00 0.00]<br>G [-0.68 -5.19 0.00 -5.36 -0.09]<br>T [-1.29 -5.19 -5.52 -5.36 -0.27]	A [17{108 0 0}20]<br>C [43 0 0 108 36]<br>G [35 0 108 0 29]<br>T [13 0 0 0 23]	[13]

Table S1 Position weight matrices (PWMs) and position count matrices (PCMs)
PCMs were derived experimentally from binding sites. The curly brackets in the first rows highlight the core motif. The `-`i`-`-notation indicates that i arbitrary nucleotides can occur between two subelements. *: The PCM for Bridge was derived for this study from the 17 of 27,232 sequences in EPDnew, version 005 that matched consensus sequence `CGANC-7-WYGT` at precisely position +18 with perfect spacing between sub-elements and up to one mismatch allowed.

			Consensus sequence			PWM		
**CPE**	**Expected location**	**Functional window**	**Sequence**	**Accepted mismatches**	q	**Cutoff**	q	
TATA box	-31	[-33,-29]	TATAWAAR	0	0.01%	0.939	0.01%	
			(symbols: 8)	1	0.19%	0.855	0.19%	
				···	···	0.790	1.15%	*
				2	2.04%	0.766	2.03%	
BREu	-38	[-40,-36]	SSRCGCC	0	0.65%	0.926	0.65%	
			(symbols: 7)	1	8.06%	0.831	8.03%	**
				2	36.12%	0.719	36.02%	
BREd	-23	[-25,-21]	RTDKKKK	0	2.29%	0.896	2.27%	
			(symbols: 7)	1	17.22%	0.793	17.22%	**
				2	49.90%	0.689	49.81%	
Inr	-2	[-4,1]	YYANWYY	0	2.51%	0.938	2.51%	
			(symbols: 7)	···	···	0.814	18.57%	*
				1	23.05%	0.772	23.12%	
				2	66.64%	0.632	66.70%	
TCT	-1	[-3,2]	YCTYTYY	0	0.36%	0.880	0.36%	
			(symbols: 7)	1	4.79%	0.780	4.79%	**
				2	23.62%	0.679	23.65%	
XCPE1	-8	[-10,-6]	DSGYGGRASM	0	0.08%	0.891	0.08%	
			(symbols: 10)	1	1.41%	0.786	1.41%	**
				2	9.84%	0.677	9.80%	
XCPE2	-9	[-11,-7]	VCYCRTTRCMY	0	0.01%	0.960	0.01%	
			(symbols: 11)	1	0.27%	0.859	0.27%	
				2	2.55%	0.761	2.55%	**
DPE	28	[26,30]	RGWYV	0	12.13%	0.862	12.04%	**
			(symbols: 5)	1	66.44%	0.727	66.56%	
MTE	18	[16,20]	CSARCSSAACGS	0	0.00%	0.909	0.00%	
			(symbols: 12)	1	0.05%	0.842	0.05%	
				2	0.58%	0.776	0.58%	**
				3	3.98%	0.711	3.93%	
Bridge	18	[16,20]	CGANC-7-WYGT	0	0.03%	0.928	0.03%	
			(symbols: 9)	1	0.67%	0.851	0.68%	**
				2	6.04%	0.768	6.03%	
DCE	8	[6,10]	CTTC-6-CTGT-10-AGC	0	0.00%	0.942	0.00%	
			(symbols: 11)	1	0.00%	0.881	0.00%	
				2	0.06%	0.813	0.07%	**
				3	0.62%	0.739	0.62%	
DCE3	25	[23,27]	AGC	0	8.76%	0.709	8.76%	**
			(symbols: 3)					

Table S2 PWM parameters

Functional window refers to the 5-nucleotide range relative to the TSS at position +1 where the start position of the CPE (core motif) is expected to occur. q is the expected occurrence probability in a 5-nucleotide range in random DNA. Accepted mismatches denotes the number of non-matching symbols tolerated for a match with the consensus sequence. Cutoff denotes the lowest acceptable score for a match with the PWM (Methods). *: Biologically-justified cutoff values were available for TATA and Inr [4]. **: For the other CPEs, we chose cutoff values so that q for the PWM matched q for the consensus sequence with a set number of accepted mismatches. An exact match was required (zero mismatches) when the number of symbols in the consensus sequence was five or less. One mismatch was tolerated when the number of symbols in the sequence was between six and ten. Two mismatches were tolerated otherwise (e.g., MTE).

CPE	n	Observed	Expected	z-score	p-value (G.)	p-value (b.)
TATA box	3163	10.7%	1.4%	17.7	$< 1.2 \times 10^{-38}$ ***	$< 1.2 \times 10^{-38}$ ***
BREu	4217	14.2%	10.0%	1.7	4.07×10^{-2}	$< 1.2 \times 10^{-38}$ ***
BREd	4911	16.6%	17.4%	-1.1	0.863	1.0
Inr	12047	40.7%	19.9%	11.5	$< 1.2 \times 10^{-38}$ ***	$< 1.2 \times 10^{-38}$ ***
TCT	3482	11.8%	9.2%	3.7	1.04×10^{-4} **	$< 1.2 \times 10^{-38}$ ***
XCPE1	856	2.9%	2.5%	0.4	0.342	8.08×10^{-5} ***
XCPE2	731	2.5%	2.3%	1.1	0.136	9.84×10^{-3}
DPE	3753	12.7%	9.9%	3.6	1.73×10^{-4} **	$< 1.2 \times 10^{-38}$ ***
DPE∩Inr	1625	5.5%	4.1%	2.9	1.93×10^{-3} *	$< 1.2 \times 10^{-38}$ ***
MTE	263	0.9%	0.4%	5.4	3.95×10^{-8} ***	$< 1.2 \times 10^{-38}$ ***
MTE∩Inr	109	0.4%	0.2%	5.1	2.02×10^{-7} ***	1.41×10^{-13} ***
Bridge	638	2.2%	1.3%	3.5	2.24×10^{-4} **	$< 1.2 \times 10^{-38}$ ***
Bridge∩Inr	249	0.8%	0.5%	3.2	6.86×10^{-4} **	1.69×10^{-14} ***
DCE	656	2.2%	1.1%	3.8	7.01×10^{-5} ***	$< 1.2 \times 10^{-38}$ ***
DCE3	4145	14.0%	9.7%	4.6	2.63×10^{-6} ***	$< 1.2 \times 10^{-38}$ ***

Table S3 Localized overrepresentation of CPEs in promoter sequences
Localized overrepresentation for twelve CPEs in $N = 29{,}598$ promoter sequences as represented in the EPDnew dataset, version 006 (hg38). Observed values correspond to n/N and expected values correspond to expected occurrence probability as defined in Methods. p-values were determined according to the Gaussian (G.) and binomial (b.) distributions. DPE∩Inr: DPE was only called in sequences that contained an Inr motif (analogously for MTE∩Inr and Bridge∩Inr). *: Significant at $\alpha = 0.05$ following Bonferroni correction for twelve tests. **: Significant at $\alpha = 0.01$ following correction. ***: Significant at $\alpha = 0.001$ following correction.

CPE	n	Observed	Expected	z-score	p-value (G.)	p-value (b.)
TATA box	11903	9.4%	2.4%	22.2	$< 1.2 \times 10^{-38}$ ***	$< 1.2 \times 10^{-38}$ ***
BREu	3873	3.1%	2.8%	1.4	7.75×10^{-2}	3.03×10^{-10} ***
BREd	29391	23.2%	20.9%	2.2	1.33×10^{-2}	$< 1.2 \times 10^{-38}$ ***
Inr	45202	35.7%	22.5%	19.7	$< 1.2 \times 10^{-38}$ ***	$< 1.2 \times 10^{-38}$ ***
TCT	11529	9.1%	8.7%	0.3	0.391	4.38×10^{-7} ***
XCPE1	868	0.7%	0.7%	0.3	0.394	0.161
XCPE2	2564	2.0%	1.8%	2.1	1.62×10^{-2}	1.44×10^{-12} ***
DPE	18007	14.2%	11.4%	7.4	7.25×10^{-14} ***	$< 1.2 \times 10^{-38}$ ***
DPE∩Inr	6920	5.5%	4.1%	4.6	1.73×10^{-6} ***	$< 1.2 \times 10^{-38}$ ***
MTE	238	0.2%	0.1%	3.7	1.19×10^{-4} **	1.76×10^{-6} ***
MTE∩Inr	99	0.1%	0.1%	3.2	6.37×10^{-4} **	1.17×10^{-4} **
Bridge	926	0.7%	0.6%	3.2	7.84×10^{-4} **	2.71×10^{-10} ***
Bridge∩Inr	410	0.3%	0.2%	4.6	2.66×10^{-6} ***	8.78×10^{-14} ***
DCE	1105	0.9%	0.6%	5.4	3.22×10^{-8} ***	$< 1.2 \times 10^{-38}$ ***
DCE3	14769	11.7%	9.9%	2.9	1.66×10^{-3} *	$< 1.2 \times 10^{-38}$ ***

Table S4 Localized overrepresentation of CPEs in enhancer sequences
Localized overrepresentation for twelve CPEs in $N = 126{,}570$ transcribed enhancer sequences from the FANTOM5 dataset (hg38). p-values, DPE∩Inr, MTE∩Inr, Bridge∩Inr, *, ** and *** as in Table **S3**.

CPE	Overall	TATA$_+$	Inr$_+$	DPE$_+$	BREu$_+$
TATA$_+$	3163 (10.7%)	···	1269 (40.1%)<br>$p = 0.765$	422 (13.3%)<br>$p = 0.124$	311 (9.8%)<br>$p = 1.00$
TATA$_-$	26435 (89.3%)	···	10778 (40.8%)<br>$p = 0.247$	3331 (12.6%)<br>$p = 0.887$	3906 (14.8%) *<br>$p < 1.2 \times 10^{-38}$
Inr$_+$	12047 (40.7%)	1269 (10.5%)<br>$p = 0.765$	···	1625 (13.5%) *<br>$p = 2.92 \times 10^{-4}$	1712 (14.2%)<br>$p = 0.566$
Inr$_-$	17551 (59.3%)	1894 (10.8%)<br>$p = 0.247$	···	2128 (12.1%)<br>$p = 1.00$	2505 (14.3%)<br>$p = 0.448$
DPE$_+$∩Inr$_+$	1625 (5.5%)	184 (11.3%)<br>$p = 0.142$	···	···	204 (12.6%)<br>$p = 0.983$
DPE$_-$∩Inr$_+$	10422 (35.2%)	1085 (10.4%)<br>$p = 0.876$	···	···	1508 (14.5%)<br>$p = 2.06 \times 10^{-2}$
BREu$_+$	4217 (14.2%)	311 (7.4%)<br>$p = 1.00$	1712 (40.6%)<br>$p = 0.566$	489 (11.6%)<br>$p = 0.990$	···
BREu$_-$	25381 (85.8%)	2852 (11.2%) *<br>$p < 1.2 \times 10^{-38}$	10335 (40.7%)<br>$p = 0.448$	3264 (12.9%)<br>$p = 0.0113$	···

Table S5 Co-occurrence of CPEs in promoter sequences

The columns indicate counts of promoters (from among a total of $N = 29{,}598$ promoters) in which the indicated combination of CPEs was found. p-values were determined using Fisher's exact test followed by Bonferroni correction (Methods). *: Significant at $\alpha = 0.05$ following correction. DPE$_+$∩Inr$_+$ and DPE$_-$∩Inr$_+$: DPE was only analyzed in sequences that contained an Inr motif.

CPE	Overall	TATA$_+$	Inr$_+$	DPE$_+$	BREu$_+$
TATA$_+$	11903 (9.4%)	···	3773 (31.7%)<br>$p = 1.00$	1597 (13.4%)<br>$p = 0.996$	195 (1.6%)<br>$p = 1.00$
TATA$_-$	114667 (90.6%)	···	41429 (36.1%) *<br>$p < 1.2 \times 10^{-38}$	16410 (14.3%)<br>$p = 3.90 \times 10^{-3}$	3678 (3.2%) *<br>$p < 1.2 \times 10^{-38}$
Inr$_+$	45202 (35.7%)	3773 (8.3%)<br>$p = 1.00$	···	6920 (15.3%) *<br>$p < 1.2 \times 10^{-38}$	1530 (3.4%) *<br>$p = 3.58 \times 10^{-7}$
Inr$_-$	81368 (64.3%)	8130 (10.0%) *<br>$p < 1.2 \times 10^{-38}$	···	11087 (13.6%)<br>$p = 1.00$	2343 (2.9%)<br>$p = 1.00$
DPE$_+$∩Inr$_+$	6920 (5.5%)	504 (7.3%)<br>1.00	···	···	244 (3.5%)<br>0.250
DPE$_-$∩Inr$_+$	38,282 (30.2%)	3269 (8.5%) *<br>$p = 2.28 \times 10^{-4}$	···	···	1286 (3.4%)<br>0.772
BREu$_+$	3873 (3.1%)	195 (5.0%)<br>$p = 1.00$	1530 (39.5%) *<br>$p = 3.58 \times 10^{-7}$	558 (14.4%)<br>$p = 0.379$	···
BREu$_-$	122697 (96.9%)	11708 (9.5%) *<br>$p < 1.2 \times 10^{-38}$	43672 (35.6%)<br>$p = 1.00$	17449 (14.2%)<br>$p = 0.639$	···

Table S6 Co-occurrence of CPEs in enhancer sequences

The columns indicate counts of enhancer transcripts (from among a total of $N = 126{,}570$ enhancer transcripts) in which the indicated combination of CPEs was found. p-values, *, DPE$_+$∩Inr$_+$ and DPE$_-$∩Inr$_+$ as in Table **S5**.

	Promoters			Enhancers		
	**CGI**	**Non-CGI**	***p*-value**	**CGI**	**Non-CGI**	***p*-value**
Overall	0.285	0.783	$< 10^{-300}$	0.703	0.821	$< 10^{-300}$
	**TATA$_+$**	**TATA$_-$**	***p*-value**	**TATA$_+$**	**TATA$_-$**	***p*-value**
Overall	0.746	0.461	1.9×10^{-156}	0.843	0.816	3.1×10^{-105}
CGI	0.472	0.272	5.5×10^{-32}	0.810	0.699	9.3×10^{-10}
Non-CGI	0.854	0.761	3.7×10^{-38}	0.844	0.818	1.0×10^{-87}

Table S7 Association of CGI and TATA presence with tissue specificity (τ)
The table shows τ medians of different subsets of promoters and enhancers (e.g., CGI, TATA$_+$ or TATA$_+ \cap$CGI). p-values were calculated with the Mann–Whitney U test. The values are derived from primary cell data.

	Overall		CGI		Non-CGI	
	τ **median**	n	τ **median**	n	τ **median**	n
Overall	0.5121	28045	0.2776	17034	0.7714	11011
TATA box	0.7257	2820	0.4816	1132	0.8205	1688
BREu	0.3339	4123	0.2769	3563	0.7150	560
BREd	0.5490	4616	0.2704	2575	0.8057	2041
Inr	0.5441	11398	0.2991	6539	0.7765	4859
TCT	0.4368	3268	0.2359	2067	0.7790	1201
XCPE1	0.3857	833	0.3121	699	0.6636	134
XCPE2	0.5060	696	0.2294	431	0.8054	265
DPE	0.5520	3541	0.2757	1899	0.7732	1642
DPE∩Inr	0.5999	1533	0.3242	751	0.7783	782
MTE	0.4004	255	0.3166	214	0.7828	41
MTE∩Inr	0.3396	107	0.2350	87	0.8021	20
Bridge	0.4355	609	0.3343	473	0.6650	136
Bridge∩Inr	0.4413	240	0.3264	179	0.8333	61
DCE	0.4748	624	0.3595	442	0.7483	182
DCE3	0.5593	3948	0.3103	2458	0.7991	1490

Table S8 Tissue specificity of promoters per CPE

The table shows τ statistics for different subsets of promoters. 1553 (5.2%) of 29,598 promoter transcripts were removed because of insufficient CAGE tag coverage. The data shown here is from an analysis of the tissue libraries. Comparable results were obtained from the primary cell data (not shown). DPE∩Inr: DPE was only called in sequences that contained an Inr motif (analogously for MTE∩Inr and Bridge∩Inr).

	Overall		CGI		Non-CGI	
	τ **median**	n	τ **median**	n	τ **median**	n
Overall	0.9407	117279	0.7953	4599	0.9439	112680
TATA box	0.9610	10771	0.8776	179	0.9622	10592
BREu	0.9042	3703	0.7648	679	0.9293	3024
BREd	0.9401	27265	0.8237	675	0.9418	26590
Inr	0.9433	41896	0.8155	1668	0.9466	40228
TCT	0.9339	10780	0.7741	489	0.9374	10291
XCPE1	0.8846	836	0.7297	134	0.9097	702
XCPE2	0.9394	2386	0.7768	145	0.9436	2241
DPE	0.9441	16672	0.8108	554	0.9469	16118
DPE∩Inr	0.9447	6401	0.8407	218	0.9470	6183
MTE	0.9296	228	0.7676	29	0.9410	199
MTE∩Inr	0.9387	95	0.8730	11	0.9428	84
Bridge	0.9301	860	0.7254	86	0.9401	774
Bridge∩Inr	0.9361	381	0.8432	34	0.9415	347
DCE	0.9290	1046	0.7855	96	0.9346	950
DCE3	0.9367	13800	0.7839	990	0.9420	12810

Table S9 Tissue specificity of enhancers per CPE

The table shows τ statistics for different subsets of enhancer transcripts. 9291 (7.3%) of 126,570 enhancer transcripts were removed because of insufficient CAGE tag coverage. The data shown here is from an analysis of the tissue libraries. Comparable results were obtained from the primary cell data (not shown). DPE∩Inr, MTE∩Inr and Bridge∩Inr as in Table **S8**.

	With H3K27ac signal	Without H3K27ac signal
CGI promoters	17248 (99.5%)	88 (0.5%)
Non-CGI promoters	11957 (97.5%)	305 (2.5%)
CGI enhancers	3457 (96.4%)	130 (3.6%)
Non-CGI enhancers	53342 (89.4%)	6356 (10.6%)
Controls CGI promoters	14080 (81.2%)	3256 (18.8%)
Controls non-CGI promoters	9961 (81.2%)	2301 (18.8%)
Controls CGI enhancers	2551 (71.1%)	1036 (28.9%)
Controls non-CGI enhancers	40340 (67.6%)	19358 (32.4%)

Table S10 Analysis of overlaps of CGI- and non-CGI-associated promoters and enhancers Analysis using all H3K27ac annotations from the Ensembl regulatory build (release 98, hg38), regardless of signal strength and cell line [14]. 89.8% of FANTOM5 enhancers overlap with H3K27ac signal. We additionally chose a length-matched random genomic region as control for each CGI and non-CGI promoter and enhancer, and then counted the overlaps with at least one H3K27ac mark. It can be seen that H3K27ac is commonly encountered throughout the genome, but that the EPDnew promoter dataset and FANTOM5 enhancer dataset displayed a higher amount of overlap than corresponding random regions.

Transcription factor	Non-CGI promoters	Promoter-associated CGIs		CGI promoters	
	Peaks	Peaks	p-value	Peaks	p-value
ETS1	818 (6.68%)	7273 (42.1%)	$< 10^{-300}$	4542 (26.3%)	$< 10^{-300}$
KLF5	725 (5.92%)	6719 (38.9%)	$< 10^{-300}$	3985 (23.1%)	1.79×10^{-286}
FLI1	1298 (10.6%)	8945 (51.8%)	$< 10^{-300}$	6045 (35.0%)	$< 10^{-300}$
ZN143	286 (2.34%)	3163 (18.3%)	$< 10^{-300}$	1847 (10.7%)	5.03×10^{-164}
CTCF	2049 (16.7%)	13194 (76.4%)	$< 10^{-300}$	8824 (51.1%)	$< 10^{-300}$
E2F1	1216 (9.93%)	14593 (84.5%)	$< 10^{-300}$	9439 (54.7%)	$< 10^{-300}$
CREB1	486 (3.97%)	6313 (36.6%)	$< 10^{-300}$	3800 (22.0%)	$< 10^{-300}$
TYY1	579 (4.73%)	7439 (43.1%)	$< 10^{-300}$	4724 (27.4%)	$< 10^{-300}$
ERG	713 (5.82%)	4887 (28.3%)	$< 10^{-300}$	3578 (20.7%)	5.70×10^{-237}
HTF4	504 (4.12%)	4349 (25.2%)	$< 10^{-300}$	2817 (16.3%)	2.16×10^{-213}
MAX	1324 (10.8%)	10504 (60.8%)	$< 10^{-300}$	7052 (40.8%)	$< 10^{-300}$
GABPA	908 (7.41%)	8448 (48.9%)	$< 10^{-300}$	5063 (29.3%)	$< 10^{-300}$
ELF1	492 (4.02%)	7161 (41.5%)	$< 10^{-300}$	4331 (25.1%)	$< 10^{-300}$
ETV1	421 (3.44%)	6041 (35.0%)	$< 10^{-300}$	3483 (20.2%)	$< 10^{-300}$
SP1	1717 (14.0%)	15039 (87.1%)	$< 10^{-300}$	9767 (56.6%)	$< 10^{-300}$
NRF1	421 (3.44%)	8540 (49.5%)	$< 10^{-300}$	4955 (28.7%)	$< 10^{-300}$
E2F4	132 (1.08%)	3435 (19.9%)	$< 10^{-300}$	2001 (11.6%)	6.23×10^{-285}
AP2C	749 (6.12%)	4791 (27.7%)	$< 10^{-300}$	3035 (17.6%)	8.17×10^{-157}
ESR1	1635 (13.4%)	8314 (48.2%)	$< 10^{-300}$	6168 (35.7%)	8.28×10^{-259}
MYC	1711 (14.0%)	10386 (60.2%)	$< 10^{-300}$	8430 (48.8%)	$< 10^{-300}$
ZFX	186 (1.52%)	4165 (24.1%)	$< 10^{-300}$	2438 (14.1%)	$< 10^{-300}$
NFYA	243 (1.98%)	2714 (15.7%)	$< 10^{-300}$	1738 (10.1%)	1.29×10^{-167}
MYCN	276 (2.25%)	4594 (26.6%)	$< 10^{-300}$	2697 (15.6%)	$< 10^{-300}$
RXRA	349 (2.85%)	3342 (19.4%)	$< 10^{-300}$	2151 (12.5%)	9.48×10^{-182}
REST	281 (2.29%)	2861 (16.6%)	$< 10^{-300}$	2046 (11.9%)	5.30×10^{-198}
HIF1A	292 (2.38%)	2751 (15.9%)	$< 10^{-300}$	1656 (9.59%)	5.85×10^{-132}
ELK4	129 (1.05%)	2096 (12.1%)	$< 10^{-300}$	1121 (6.49%)	2.21×10^{-125}
P53	531 (4.34%)	3471 (20.1%)	2.90×10^{-296}	2390 (13.8%)	5.78×10^{-145}
EGR2	110 (0.90%)	1816 (10.5%)	6.79×10^{-268}	1106 (6.41%)	2.48×10^{-134}
SUH	266 (2.17%)	2198 (12.7%)	7.29×10^{-231}	1275 (7.38%)	2.35×10^{-86}
ZBT17	397 (3.24%)	2290 (13.3%)	1.05×10^{-181}	1400 (8.11%)	9.07×10^{-62}
ANDR	1313 (10.7%)	4480 (25.9%)	1.06×10^{-167}	3471 (20.1%)	8.73×10^{-77}
KLF4	208 (1.70%)	1590 (9.21%)	2.24×10^{-161}	958 (5.55%)	1.64×10^{-62}
TF65	572 (4.67%)	2532 (14.7%)	1.23×10^{-149}	1952 (11.3%)	7.75×10^{-80}
RUNX1	774 (6.32%)	3022 (17.5%)	3.62×10^{-148}	2180 (12.6%)	1.45×10^{-59}
RARA	218 (1.78%)	1538 (8.91%)	9.14×10^{-148}	1001 (5.80%)	4.84×10^{-65}
VDR	238 (1.94%)	1533 (8.88%)	3.62×10^{-137}	929 (5.38%)	9.48×10^{-49}
TAL1	480 (3.92%)	2154 (12.5%)	3.76×10^{-131}	1354 (7.84%)	2.82×10^{-38}
SPI1	1419 (11.6%)	4165 (24.1%)	2.12×10^{-116}	2859 (16.6%)	1.51×10^{-23}
BCL6	336 (2.74%)	1633 (9.46%)	4.83×10^{-111}	1018 (5.90%)	4.43×10^{-34}
STAT1	350 (2.86%)	1666 (9.65%)	1.38×10^{-110}	974 (5.64%)	7.14×10^{-27}
PRGR	503 (4.11%)	1589 (9.20%)	5.85×10^{-57}	1079 (6.25%)	1.29×10^{-12}
GCR	500 (4.08%)	1549 (8.97%)	2.02×10^{-53}	1070 (6.20%)	2.36×10^{-12}

Table S11 Promoter ChIP-seq peaks

The table lists counts and p-values of ChIP-seq peaks of individual transcription factors in promoters not associated with a CGI, in promoter-associated CGIs, and in CGI-associated promoters. There were 17,336 CGI-associated promoters, and 12,262 promoters not associated with CGIs. Percentages of items demonstrating ChIP-seq peaks are shown in parentheses. p-values were calculated versus non-CGI promoters with Fisher's exact test using Bonferroni correction.

Transcription factor	Non-CGI enhancers	Enhancer-associated CGIs		CGI enhancers	
	Peaks	Peaks	p-value	Peaks	p-value
NRF1	358 (0.60%)	512 (14.3%)	$< 10^{-300}$	468 (13.0%)	$< 10^{-300}$
CTCF	6959 (11.7%)	1787 (49.8%)	$< 10^{-300}$	1697 (47.3%)	$< 10^{-300}$
SP1	2616 (4.38%)	1931 (53.8%)	$< 10^{-300}$	1805 (50.3%)	$< 10^{-300}$
E2F1	1414 (2.37%)	1466 (40.9%)	$< 10^{-300}$	1347 (37.6%)	$< 10^{-300}$
MYC	7091 (11.9%)	1543 (43.0%)	4.21×10^{-285}	1527 (42.6%)	1.38×10^{-278}
MAX	5580 (9.35%)	1246 (34.7%)	4.68×10^{-246}	1188 (33.1%)	7.35×10^{-222}
MYCN	503 (0.84%)	411 (11.5%)	3.34×10^{-244}	387 (10.8%)	9.88×10^{-224}
CREB1	673 (1.13%)	385 (10.7%)	2.06×10^{-189}	355 (9.90%)	1.42×10^{-166}
KLF5	1858 (3.11%)	533 (14.9%)	2.58×10^{-151}	514 (14.3%)	6.09×10^{-141}
ESR1	7109 (11.9%)	1099 (30.6%)	4.90×10^{-125}	1083 (30.2%)	4.12×10^{-120}
GABPA	2417 (4.05%)	554 (15.4%)	1.64×10^{-122}	504 (14.1%)	1.89×10^{-99}
FLI1	5672 (9.50%)	934 (26.0%)	1.16×10^{-121}	931 (26.0%)	1.31×10^{-120}
ETS1	2277 (3.81%)	494 (13.8%)	9.13×10^{-103}	456 (12.7%)	1.02×10^{-85}
AP2C	3633 (6.09%)	550 (15.3%)	5.64×10^{-65}	540 (15.1%)	1.32×10^{-61}
HTF4	2045 (3.43%)	372 (10.4%)	1.65×10^{-61}	360 (10.0%)	1.44×10^{-56}
RXRA	1113 (1.86%)	222 (6.19%)	9.57×10^{-43}	211 (5.88%)	7.24×10^{-38}
RARA	713 (1.19%)	163 (4.54%)	1.27×10^{-37}	154 (4.29%)	3.65×10^{-33}
HIF1A	991 (1.66%)	192 (5.35%)	1.28×10^{-35}	198 (5.52%)	2.58×10^{-38}
JUN	5178 (8.67%)	113 (3.15%)	2.96×10^{-32}	135 (3.76%)	3.31×10^{-24}
ERG	3389 (5.68%)	415 (11.6%)	2.15×10^{-31}	427 (11.9%)	1.42×10^{-34}
ZBT17	958 (1.60%)	174 (4.85%)	2.02×10^{-29}	178 (4.96%)	3.98×10^{-31}
P53	2768 (4.64%)	339 (9.45%)	6.85×10^{-26}	336 (9.37%)	3.85×10^{-25}
KLF4	743 (1.24%)	136 (3.79%)	2.49×10^{-23}	143 (3.99%)	2.16×10^{-26}
FOS	3485 (5.84%)	80 (2.23%)	2.78×10^{-20}	101 (2.82%)	3.28×10^{-13}
FOSL2	1376 (2.30%)	18 (0.50%)	1.49×10^{-14}	22 (0.61%)	2.73×10^{-12}
CEBPB	3252 (5.45%)	88 (2.45%)	3.36×10^{-14}	110 (3.07%)	3.88×10^{-8}
PO5F1	1100 (1.84%)	145 (4.04%)	1.84×10^{-13}	144 (4.01%)	4.72×10^{-13}
JUND	2869 (4.81%)	76 (2.12%)	5.07×10^{-13}	89 (2.48%)	3.62×10^{-9}
AP2A	1018 (1.71%)	136 (3.79%)	5.47×10^{-13}	143 (3.99%)	2.79×10^{-15}
CEBPA	2209 (3.70%)	58 (1.62%)	4.34×10^{-10}	76 (2.12%)	4.75×10^{-5}
ANDR	7349 (12.3%)	609 (17.0%)	1.00×10^{-9}	629 (17.5%)	3.67×10^{-12}
GATA3	2131 (3.57%)	57 (1.59%)	2.03×10^{-9}	69 (1.92%)	6.03×10^{-6}
GATA2	3113 (5.21%)	99 (2.76%)	2.36×10^{-9}	112 (3.12%)	2.45×10^{-6}
TEAD1	2091 (3.50%)	56 (1.56%)	3.57×10^{-9}	75 (2.09%)	4.50×10^{-4}
PAX5	597 (1.00%)	84 (2.34%)	8.46×10^{-9}	85 (2.37%)	2.97×10^{-9}
MITF	875 (1.47%)	107 (2.98%)	3.65×10^{-8}	105 (2.93%)	1.36×10^{-7}
VDR	1183 (1.98%)	128 (3.57%)	8.06×10^{-7}	131 (3.65%)	1.87×10^{-7}
SUH	1197 (2.01%)	129 (3.60%)	9.64×10^{-7}	121 (3.37%)	5.24×10^{-5}
SMAD1	572 (0.96%)	70 (1.95%)	3.88×10^{-5}	64 (1.78%)	1.74×10^{-3}
FOXA2	979 (1.64%)	104 (2.90%)	3.93×10^{-5}	109 (3.04%)	3.00×10^{-6}
FOXA1	5460 (9.15%)	235 (6.55%)	6.03×10^{-5}	277 (7.72%)	9.18×10^{-1}
BCL6	1904 (3.19%)	172 (4.80%)	2.34×10^{-4}	185 (5.16%)	1.07×10^{-6}

Table S12 Enhancer ChIP-seq peaks

The table lists counts and p-values of ChIP-seq peaks of individual transcription factors in enhancers not associated with a CGI, in enhancer-associated CGIs, and in CGI-associated enhancers. There were 3587 CGI-associated enhancers, and 59,698 enhancers not associated with CGIs. Percentages of items demonstrating ChIP-seq peaks are shown in parentheses. p-values were calculated versus non-CGI enhancers with Fisher's exact test using Bonferroni correction.

GO term		CGI enhancers	Population	p-value
GO:0140110	transcription regulator activity	633/2741 (23.1%)	1806/11578 (15.6%)	7.81×10^{-29}
GO:0005694	chromosome	587/2741 (21.4%)	1651/11578 (14.3%)	1.36×10^{-17}
GO:1901363	heterocyclic compound binding	1160/2741 (42.3%)	3966/11578 (34.3%)	1.05×10^{-13}
GO:0097159	organic cyclic compound binding	1168/2741 (42.6%)	4018/11578 (34.7%)	8.63×10^{-13}
GO:0031974	membrane-enclosed lumen	1060/2741 (38.7%)	3789/11578 (32.7%)	2.99×10^{-11}
GO:0046483	heterocycle metabolic process	1105/2741 (40.3%)	3812/11578 (32.9%)	9.07×10^{-11}
GO:1901360	organic cyclic compound metabolic process	1125/2741 (41.0%)	3954/11578 (34.2%)	6.59×10^{-10}
GO:0006725	cellular aromatic compound metabolic process	1107/2741 (40.4%)	3838/11578 (33.1%)	6.63×10^{-10}
GO:0005488	binding	2273/2741 (82.9%)	9149/11578 (79.0%)	4.79×10^{-8}
GO:0001067	regulatory region nucleic acid binding	306/2741 (11.2%)	747/11578 (6.50%)	7.32×10^{-7}
GO:0009058	biosynthetic process	1128/2741 (41.2%)	4031/11578 (34.8%)	7.33×10^{-7}
GO:0003677	DNA binding	618/2741 (22.5%)	1741/11578 (15.0%)	2.26×10^{-6}
GO:0034641	cellular nitrogen compound metabolic process	1157/2741 (42.2%)	4090/11578 (35.3%)	9.79×10^{-6}
GO:0043226	organelle	2027/2741 (74.0%)	8115/11578 (70.1%)	1.36×10^{-5}
GO:0010467	gene expression	1007/2741 (36.7%)	3510/11578 (30.3%)	5.19×10^{-5}
GO:0005634	nucleus	1254/2741 (45.7%)	4527/11578 (39.1%)	6.24×10^{-5}
GO:0043228	non-membrane-bounded organelle	1006/2741 (36.7%)	3610/11578 (31.2%)	4.16×10^{-4}

Table S13 Gene Ontology (GO) analysis of genes regulated by CGI-associated enhancers
GO terms characterizing genes (n = 2743) that display evidence of regulation by CGI-associated enhancers. Significant GO terms with a p-value less than 0.001 are shown. The set of genes associated with non-CGI enhancers (n = 11,138) did not have any significant GO terms. The population set was defined as the union of the CGI- and non-CGI-associated sets (n = 13,881 genes). Overrepresentation analysis by the parent-child intersection method of the Ontologizer [15, 16]. The population set, defined as the union of the four sets, contained 13,668 genes. The gene counts shown in the table refer to the number of mappable genes with GO annotations. The enhancer-gene associations were derived from the FANTOM5 file: `http://slidebase.binf.ku.dk/human_enhancers/presets/serve/enhancer_tss_associations`

References

[1] Dreos, R., Ambrosini, G., Groux, R., Cavin Périer, R., and Bucher, P. (January, 2017) The eukaryotic promoter database in its 30th year: focus on non-vertebrate organisms. *Nucleic acids research,* **45**, D51–D55.

[2] Arner, E., Daub, C. O., Vitting-Seerup, K., Andersson, R., Lilje, B., Drabløs, F., Lennartsson, A., Rönnerblad, M., Hrydziuszko, O., Vitezic, M., et al. (February, 2015) Transcribed enhancers lead waves of coordinated transcription in transitioning mammalian cells. *Science (New York, N.Y.),* **347**, 1010–1014.

[3] Vorontsov, I. E., Fedorova, A. D., Yevshin, I. S., Sharipov, R. N., Kolpakov, F. A., Makeev, V. J., and Kulakovskiy, I. V. (October, 2018) Genome-wide map of human and mouse transcription factor binding sites aggregated from ChIP-Seq data. *BMC research notes,* **11**, 756.

[4] Bucher, P. (April, 1990) Weight matrix descriptions of four eukaryotic RNA polymerase II promoter elements derived from 502 unrelated promoter sequences. *Journal of molecular biology,* **212**, 563–578.

[5] Lagrange, T., Kapanidis, A. N., Tang, H., Reinberg, D., and Ebright, R. H. (1998) New core promoter element in RNA polymerase-II dependent transcription: sequence-specific DNA binding by transcription factor IIB. *Genes & Development,* **12**, 34–44.

[6] Deng, W. and Roberts, S. G. E. (2005) A core promoter element downstream of the TATA box that is recognized by TFIIB. *Genes & Development,* **19**(20), 2418–2423.

[7] Parry, T. J., Theisen, J. W. M., Hsu, J. Y., Wang, Y. L., Corcoran, D. L., Eustice, M., Ohler, U., and Kadonaga, J. T. (2010) The TCT motif, a key component of an RNA polymerase II transcription system for the translational machinery. *Genes and Development,* **24**(18), 2013–2018.

[8] Tokusumi, Y., Ma, Y., Song, X., Jacobson, R. H., and Takada, S. (2007) The new core promoter element XCPE1 (X Core Promoter Element 1) directs activator-, mediator-, and TATA-binding protein-dependent but TFIID-independent RNA polymerase II transcription from TATA-less promoters. *Molecular and cellular biology,* **27**(5), 1844–58.

[9] Anish, R., Hossain, M. B., Jacobson, R. H., and Takada, S. (2009) Characterization of transcription from TATA-less promoters: Identification of a new core promoter element XCPE2 and analysis of factor requirements. *PLoS ONE,* **4**(4).

[10] Kutach, A. K. and Kadonaga, J. T. (2000) The downstream promoter element DPE appears to be as widely used as the TATA box in Drosophila core promoters. *Molecular and cellular biology,* **20**(13), 4754–4764.

[11] Lim, C. Y., Santoso, B., Boulay, T., Dong, E., Ohler, U., and Kadonaga, J. T. (2004) The MTE , a new core promoter element for transcription by RNA polymerase II. *Genes and Development,* **32**, 1606–1617.

[12] Theisen, J. W. M., Lim, C. Y., and Kadonaga, J. T. (2010) Three key subregions contribute to the function of the downstream RNA polymerase II core promoter. *Molecular and cellular biology,* **30**(14), 3471–3479.

[13] Lee, D.-H., Gershenzon, N., Gupta, M., Ioshikhes, I. P., Reinberg, D., and Lewis, B. A. (2005) Functional Characterization of Core Promoter Elements: the Downstream Core Element Is Recognized by TAF1. *Molecular and cellular biology,* **25**(21), 9674–9686.

[14] Zerbino, D. R., Achuthan, P., Akanni, W., Amode, M. R., Barrell, D., Bhai, J., Billis, K., Cummins, C., Gall, A., Girón, C. G., et al. (January, 2018) Ensembl 2018. *Nucleic acids research,* **46**, D754–D761.

[15] Grossmann, S., Bauer, S., Robinson, P. N., and Vingron, M. (November, 2007) Improved detection of overrepresentation of Gene-Ontology annotations with parent child analysis. *Bioinformatics (Oxford, England),* **23**, 3024–3031.

[16] Bauer, S., Grossmann, S., Vingron, M., and Robinson, P. N. (July, 2008) Ontologizer 2.0–a multifunctional tool for GO term enrichment analysis and data exploration. *Bioinformatics (Oxford, England),* **24**, 1650–1651.
